# Loss of endothelial cell FAK represses cisplatin-induced vascular senescence in distal niches to reduce lung metastatic burden

**DOI:** 10.21203/rs.3.rs-10046163/v1

**Published:** 2026-08-25

**Authors:** Madeleine Benguigui, Abby Lockwood, Rochani Rajendram, Turkan Gizer, Edward Carter, Eleni Maniati, Pedro Casado, Vinothini Rajeeve, Gilbert Fruhwirth, Cameron Lang, Juan Pedro Martinez-Barbera, Scott Haston, Kairbaan Hodivala-Dilke, Ana Rita Pedrosa

**Affiliations:** Barts Cancer Institute; Barts Cancer Institute; Barts Cancer Institute; Barts Cancer Institute; University of Bath; Bats Cancer Institute; Institute of Cancer, Queen Mary University of London; Barts Cancer Institute - Queen Mary University of London; King’s College London; Comprehensive Cancer Centre, School of Cancer and Pharmaceutical Sciences, King’s College London,; University College London; University College London; Barts Cancer Institute; Barts Cancer Institute

**Keywords:** endothelial cells, focal adhesion kinase, cisplatin, senescence, metastasis

## Abstract

Platinum-based chemotherapy is widely used to treat non-small cell lung cancer: however, intrathoracic metastasis remains a major clinical challenge associated with increased morbidity. While chemotherapy is generally viewed as acting directly on tumour cells, it can also reprogramme the tumour microenvironment, including vascular endothelial cells. Here, we identify endothelial cell focal adhesion kinase (FAK) as a regulator of cisplatin-induced vascular stress responses that promote metastatic seeding in the lung. Using inducible endothelial cell-specific FAK loss- and endothelial cell-specific kinase-dead mouse models, we show that endothelial cell FAK is required for cisplatin-enhanced lung metastasis. In the pre-metastatic lung, cisplatin induces endothelial cell DNA damage and FAK-dependent transcriptional reprogramming enriched for p53-responsive, stress-adaptive, and senescence-associated genes. Cisplatin treatment also promotes an endothelial cell FAK-dependent secretory and adhesive state increasing tumour cell binding and metastasis seeding in the lung. Mechanistically, cisplatin triggers a transient nuclear accumulation of FAK, where kinase activity supports early ATM signaling and DNA repair. Overall, our data indicate that targeting endothelial cell FAK suppresses these responses and reduces cisplatin-induced lung metastasis.

## Summary statement

This study shows that cisplatin can prime lung endothelial cells for enhanced metastasis. Endothelial cell FAK drives this response by coupling DNA-damage to vascular stress signalling and senescence-like remodelling, highlighting a stromal mechanism that may be targeted to reduce lung metastasis.

## Introduction

Lung cancer remains the leading cause of cancer mortality worldwide^1^. Most patients receive cytotoxic chemotherapy, commonly including platinum-based therapies such as cisplatin, as first-line treatment. However, resistance is common and primary tumour relapse and metastasis ultimately are the main causes of cancer-related deaths^2^. Intrathoracic metastasis, which includes malignant pleural or pericardial effusion/nodules and contralateral lung nodules, represent a major clinical challenge due to its association with stage IV disease and poor survival^3,4^. In NSCLC, chemoresistance arises from tumour-intrinsic (e.g., enhanced DNA repair, EMT, stemness) in combination with tumour-extrinsic mechanisms in the tumour microenvironment (TME) including cellular senescence^5,6^. Overcoming chemoresistance in advanced NSCLC will therefore require strategies that simultaneously target cancer cells and the surrounding stromal components^7^.

Among stromal elements, blood vessel are recognized for delivering blood and oxygen^8^. Cells that line blood vessels, namely endothelial cells and blood vessel supporting mural cells, are known to also have paracrine functions that can influence nearby malignant and tumour microenvironmental cells^9–14^. Indeed, emerging evidence indicate that EC signalling can regulate therapy responses^15,16^ and thus understanding the underlying mechanisms can offer deeper insights into the control of chemoresistance ^10,13,15–17^.

Focal adhesion kinase (FAK) is a non-receptor tyrosine kinase that integrates signals from integrins and growth-factor receptors to regulate cell survival, inflammation, and cellular stress responses^18^. In the TME, endothelial cell FAK can translate these signals into vascular-derived cues that shape how tumours respond to cytotoxic therapy. We showed previously that EC-specific deletion of FAK sensitises tumour cells to DNA-damaging therapies (including doxorubicin and radiation), reducing tumour growth in mice without impairing vascular function, implicating EC-FAK in therapy response^19^. Mechanistically, EC-FAK drives NF-kB signalling and therapy-induced cytokine secretion, consistent with an angiocrine route by which the endothelium modulates chemosensitivity. Building on this, kinase-dead EC-FAK enhances doxorubicin chemosensitization while vessel numbers, perfusion, and drug delivery are unchanged, strengthening the case for a FAK-kinase dependent angiocrine mechanism^20^. Translationally, low endothelial pY397-FAK associates with greater benefit from adjuvant radiotherapy in a randomized breast cancer cohort, linking EC-FAK activation status to treatment outcomes in breast cancer patients ^21^. Similarly, in neoadjuvant-treated breast cancer, lower EC-pY397-FAK correlated with chemotherapy sensitivity and improved survival, further validating the clinical relevance of this endothelial axis^22^.

FAK regulates cellular stress mechanisms and inflammatory signalling during therapy^23,24^, and these pathways can also promote cellular senescence, a common response to DNA damage in the tumour microenvironment^25^. Cellular senescence is a stable growth arrest induced by stress including cancer therapy; in this context, termed therapy-induced senescence (TIS), affecting not only tumour cells but also cells of the tumour microenvironment^25^. Most cancer therapies can trigger senescence, and senescent cells actively remodel the tumour microenvironment through the senescence-associated secretory phenotype (SASP), promoting tumour progression, metastasis, and resistance to treatment^26^. Consequently, senotherapies, including senolytics that eliminate senescent cells and senomorphics that suppress the SASP, are being explored as combination strategies to standard cancer therapies^27^. However, their clinical translation remains challenging because senescence is highly context-, cell type- and therapy-dependent, and senescent cells can exert both tumour-suppressive and tumour-promoting effects. Defining which senescent cell populations are detrimental, when they emerge after therapy, and how they interact with other cells in the tumour microenvironment will therefore be essential for safely and effectively exploiting senescence-targeted therapies.

Senescence and focal adhesion signalling are closely connected: senescent endothelial cells acquire a larger and more flattened morphology, increased actin stress fibre formation, enhanced focal adhesion assembly and altered mechanical properties, including increased cellular stiffness^28^. As FAK is a central regulator of integrin-dependent focal adhesion dynamics and mechanotransduction, these changes are likely to intersect with FAK-dependent signalling. However, whether FAK is required to establish or maintain this senescence-associated adhesive and mechanical phenotype remains context-dependent and requires direct experimental validation.

EC senescence has also been shown to promote lung metastasis; for example, endothelial Notch1 activation induces a senescence-like EC programme that enhances metastatic dissemination to the lungs^29^. Furthermore, endothelial signals, mediated by FAK, can preconfigure metastatic niches and direct cancer-cell homing^30^. We have also shown that suppression of endothelial FAK in pancreatic ductal adenocarcinoma reduced liver and lung metastasis, improving survival in gemcitabine-treated mice and low endothelial-cell FAK levels in gemcitabine-treated patients were associated with better survival and fewer relapses^31^.

However, the consequences of DNA-damaging therapies on the pre-metastatic endothelium, and the potential involvement of endothelial cell FAK in these changes, remain to be defined. We hypothesise that DNA-damaging chemotherapy induces endothelial cell senescence, thereby generating a pro-metastatic vascular niche that facilitates tumour-cell seeding and colonisation in the lung, and that endothelial cell FAK is a key upstream regulator of this therapy-induced senescence programme and its subsequent pro-metastatic effects.

## Materials and Methods

### Ethics

All animal studies were approved by Queen Mary University London Animal Ethics Committee licensed under the UK Home Office regulations and the Guidance for the Operation of Animals (Scientific Procedures) Act 1986 (Home Office, London, UK), including Amendment Regulations 2012 and UK Coordinating Committee on Cancer Research Guidelines for the Welfare and Use of Animals in Cancer Research.

### Mice

*Pdgfb-iCre*^*ERT*^*;FAK*^*fl/fl*^*, Pdgfb-iCre*^*ERT*^*;FAK*^*fl/fl*^*;R26*^*K454R/K454R*^ and respective control mice (*FAK*^*fl/f*l^and *FAK*^*fl/fl*^*; R26*^*K454R/K454R*^) were maintained on a pure C57BL/6 background. Both male and female mice were used, with minimum starting body weights of 22 g and 20 g, respectively. Details of the floxing strategy used in this model have been described previously^32,33^. *FAK*^*fl/fl*^*; R26*^*K454R/K454R*^ have been reported to have comparable total FAK expression relative to WT mice^32^. *Pdgfb-iCre*^*ERT*^*; FAK*^*fl/fl*^ mice were crossed with *p16*^*INK4A*^*- FDR*^34^ mice to generate *Pdgfb-iCre*^*ERT*^*; FAK*^*fl/fl*^*; p16*^*INK4A*^*- FDR* mice. For genetically modified unifocal lung cancer model *FSF-Kras*^*G12D*^*;Trp53*^*frt/frt* 35^ (KP) mice were crossed with *Pdgfb-iCre*^*ERT*^*; FAK*^*fl/fl*^ to generate *FSF-Kras*^*G12D*^*; Trp53*^*frt/frt*^ ; *Pdgfb-iCre*^*ERT*^*; FAK*^*fl/fl*^ mice.

For in-house–bred animals, quarterly health screening was performed in accordance with FELASA guidelines (https://felasa.eu/Portals/1/WorkingGroupsPublic/TORs_MouseHealth_Monitoring_Methodology%20VF.pdf?ver=OF6rqWUG0aubICoce4r08A%3d%3d) to confirm the absence of known pathogens; no clinical abnormalities were detected. Mice were housed in groups of four to five per individually ventilated cage under a 12-h light/dark cycle (lights on 06:30–18:30) with controlled temperature (21 ± 1 °C) and relative humidity (40–60%). Cages contained 1–1.5 cm of bedding and environmental enrichment, including cardboard tunnel boxes and crinkled paper nesting material. Animals had ad libitum access to food and water.

### Cell culture

The following non-small cell lung cancer (NSCLC) cell lines were used: KP cells (KPB6 RRID:CVCL_C0RJ), a kind gift from Julian Downward’s lab, derived from *LSL-Kras*^*G12D*^*;Trp53* ^*fl/fl*^ strain; Lewis lung carcinoma (LLC) cells (3 LL RRID:CVCL_5653), Catalogue No.: CRL-1642 (ATCC, Glasgow, Scotland, UK). Cell lines were mycoplasma tested (LT07–710 MycoAlert PLUS detection kit and LT07–518 MYCOALERT ASSAY CONTROL SET-10, Lonza Bioscience). KPs and LLCs were cultured in Dulbecco’s Modified Eagle Serum (DMEM) medium with 4.5% Glucose (cat. no.41966029, Gibco, Thermo Fisher scientific, UK) supplemented with 10% FBS (Gibco, cat. no. 10438026) and 1% Pen/Strep (#15140–122 Gibco) at 37°C in a 5% CO2 humidified incubator.

For animal transplantation, cells were resuspended in PBS (without Ca++/Mg++, #14190144, Gibco, ThermoFisher Scientific) as indicated in the tumour growth section below. Cells were kept on ice prior to injections.

Human pulmonary microvascular endothelial cells (HPMECs, #C-12281, PromoCell) were maintained in 0.2% porcine gelatin coated T75 flasks with endothelial basal medium MV2 with SupplementMix c-39226 (#C-22221 PromoCell) and 1% Pen/Strep (#15140–122 Gibco) at 37°C in a 5% CO2 humidified incubator. Human umbilical cord vascular endothelial cells (HUVECs, #C-12205, PromoCell) were similarly adhered with 0.2% porcine gelatin in T75 flasks with endothelial cell growth medium 2 with SupplementMix C-39216 (#C-22011 PromoCell) and 1% Pen/Strep at 37°C in a 5% CO2 humidified incubator. For all *in vitro* experiments, endothelial cell growth medium MV with SupplementMix C-39225 (#C 22020 PromoCell) and 1% Pen/Strep was used.

### Sodium Iodide symporter (mNIS) reporter

All *in vivo* traceable cell lines (LLC and KP) have been genetically engineered to stably express a murine NIS fused C-terminally to enhanced green fluorescent protein carrying the monomerising A206K mutation (mNIS-GFP). This approach has previously been shown to permit cancer cell tracking with excellent sensitivity on the whole-body level while being quantitative and non-invasive^36–38^. In brief, lung cancer cell lines were transduced with lentiviruses carrying the mNIS-GFP transgene under control of a spleen focus-forming promoter, purified (from parental non-transduced cancer cells) by fluorescence-activated cell sorting, and characterized using established methodology assessing reporter expression, subcellular localization, and function (by radiotracer Tc-99m-pertechnetate uptake)^39^.

### Tumour growth, metastasis and *in vivo* Cisplatin treatments

**For subcutaneous tumour growth experiments**, 0.5 ×10^6^ mouse LLC cells (in 100 μl of PBS) were injected subcutaneous in the flank of *Pdgfb-iCre*^*ERT+*^*;FAK*^*fl/fl*^*, Pdgfb-iCre*^*ERT+*^*;FAK*^*fl/fl*^*;R26*^*K454R/K454R*^ and respective control mice (*Pdgfb-iCre*^*ERT*-^; *FAK*^*fl/f*l^and *Pdgfb-iCre*^*ERT*-^*FAK*^*fl/fl*^*; R26*^*K454R/K454R*^). Tumour growth was measured using callipers three times a week. Once tumours reached 25–50mm^3^, all mice were given two i.p. injections of tamoxifen (150 ml of 10 mg/ml; Sigma Aldrich, St. Louis, MO, USA; T5648) 24 h apart and placed on a tamoxifen-containing diet from the second injection until the end of the experiment to induce Cre activation. Tamoxifen induction in *Pdgfb-iCre*^*ERT+*^*;FAK*^*fl/fl*^*, Pdgfb-iCre*^*ERT+*^*;FAK*^*fl/fl*^*;R26*^*K454R/K454R*^ resulted in EC-FAK deletion or EC-FAK-kinase dead mutation respectively. Tamoxifen treatment of *Pdgfb-iCre*^*ERT*-^;*FAK*^*fl/fl*^*, Pdgfb-iCre*^*ERT*-^;*FAK*^*fl/fl*^*;R26*^*K454R/K454R*^ had no effect on FAK expression and providedEC-FAK-wild type (EC-FAK WT) controls. At 24 h after the second tamoxifen injection, mice were administered three 4 mg/kg Cisplatin (Amarox, PL 49445/0179) or saline treatments (i.p.) 24–48 h apart. Animals were then either euthanised 1 day after the last Cisplatin treatment to collect primary tumour and pre-metastatic lungs, or the primary tumour was resected (mean volumes 150–200 mm^3^) under local anesthetic (Xylazin or Rompun at 10 mg/Kg, Bayer, UK, with Ketamin or Narketan at 100 mg/Kg, Vetoquinol Ltd, UK) and pre- and post-surgical analgesia (Vetergesic- 0.1 mg/Kg, Ceva Animal Health Ltd, UK). After resections, mice remained alive to assess metastasis development (3–6 weeks post-resections). For metastasis quantification (absence or presence) only mice that had at least around 150 mm^3^ at resection were analysed. Mice were culled if tumours reached the size limit or ulcerated or mice showed signs of distress, according to the humane end points established in our animal project license.

### Lung intralobular unifocal autochthonous mouse model^39^

#### Virus preparation-

Matrigel was thawed at 4 C overnight and stock virus vials were thawed on ice on the day of the experiment. Each mouse received 2.5 × 10^7^ PFU of adenovirus- FlpO (Ad5CMVFlpO, VVC-U of Iowa 530, University of Iowa, Iowa, USA) in a final volume of 10 μl (5 μl high concentration Matrigel, Catalogue No.: 354263/ 5 μl Minimum essential medium- MEM, Catalogue No.: 21090–022, Gibco, ThermoFisher Scientific, Altrincham, Cheshire, UK). MEM/virus mixture was prepared by adding 2 mM L-glutamine and 12 mM CaCl2. Virus was added to modified MEM and mixed by inverting the tube. Once prepared, virus/MEM mixture is mixed carefully with an equal volume of Matrigel to avoid bubbles and kept on ice before injection.

#### Thoracotomy procedure-

In brief, mice were anaesthetised and laid on their right side, and the pre-shaved left thoracic area was sterilised. The ribcage was identified and a vertical incision made on the mid-point between the ribcage and the shoulder. Subcutaneous tissue was removed to expose the pleura with the ribcage and left lung lobe underneath. A preloaded insulin syringe (U-100 Insulin 0.5 ml, 0.33mm (29G) ×12,7mm, #324892, BD Microfine, SLS) was then immediately inserted at a depth of 5 mm and the mixture dispensed. A cotton bud was applied to the place of injection before slowly removing the syringe. The two skin flaps were then brought together and two wound clips applied. The mouse was then laid on its left side to recover from anaesthesia. Pre- and post-surgical analgesia (Vetergesic- 0.1 mg/Kg, Ceva Animal Health Ltd, UK) was given. Further details on the intralobular unifocal method have been published previoulsy^39^.

#### Treatment schedule in KP unifocal model-

Following virus injection, mice are allowed to develop primary tumours in the left lung lobe for 6 weeks. At which point primary tumour volume is measured by CT imaging (detailed bellow), and again every 3 weeks until 18 weeks post-injection. At week 6, and once tumours become established (25–50 mm^3^), mice are randomized according to tumour volumes into 4 groups: Control (saline) KP *Pdgfb-iCre*^*ERT*-^; *FAK*^*fl/fl*^, *Pdgfb-iCre*^*ERT+*^*; FAK*^*fl/fl*^ and Cisplatin (4 mg/kg) KP *Pdgfb-iCre*^*ERT*-^; *FAK*^*fl/fl*^, *Pdgfb-iCre*^*ERT+*^*; FAK*^*fl/fl*^. At week 7, all mice were given two i.p. injections of tamoxifen (150 μl of 10 mg/ml; Sigma Aldrich, St. Louis, MO, USA; T5648) 24 h apart and placed on a tamoxifen-containing diet from the second injection until the end of the experiment to induce Cre activation. Tamoxifen induction in KP *Pdgfb-iCre*^*ERT+*^*;FAK*^*fl/fl*^*,* resulted in EC-FAK deletion. Tamoxifen treatment of KP *Pdgfb-iCre*^*ERT*-^;*FAK*^*fl/fl*^ had no effect on FAK expression and providedKPEC-FAK-wild type (KP EC-FAK WT) controls. At 24 h after the second tamoxifen injection, mice were administered 4× 4 mg/kg Cisplatin (Amarox, PL 49445/0179) or Saline treatments (i.p.) 1 week apart, for a total of 4 weeks, and left to assess survival.

**For experimental IV metastasis experiments**, following the final cisplatin administration, *Pdgfb-iCre*^*ERT*^*; FAK*^*fl/fl*^ mice were injected intravenously via the tail vein with 5 × 10^5^ pre-labelled LLC cells suspended in PBS (total volume 0.1 mL per mouse). LLC cells were labelled prior to injection using the CellTrace^™^ Violet Cell Proliferation Kit (Thermo Fisher Scientific, #C34571) according to the manufacturer’s instructions. To assess early homing, seeding, and metastatic colonisation, lungs were collected at 2 h, 48 h, and 7 days post-injection, respectively for downstream flow cytometry (FACS) analysis.

### Single-photon computed tomography (SPECT) /Computed Tomography (CT) imaging

Mice with LLC mNIS-GFP (mouse sodium iodide symporter) cell- induced subcutaneous tumours were injected via tail vein injection with 30 MBq Tc99m-pertechnetate (Technetium 99, Barts Health Radiopharmacy, Barts Health NHS Trust, London, UK) in 200 μl PBS. Following a 50-min uptake period, the mice were anaesthetised using isoflurane maintained at 300 ml/min and 2% oxygen and placed in a heated imaging bed. Respiration and anaesthesia were monitored and adjusted if necessary to maintain the breathing rate between 40 and 60 breaths per min.

Whole-body images were acquired using a multi-modal SPECT/CT scanner (VECTor^6^CT^XUHR^, MiLabs Utrecht, Houten, the Netherlands) fitted with a 0.6 mm multi-pinhole collimator (GP-M) over 30 minutes. This was followed by a CT acquisition with the following parameters: scan angle = 360°, step angle = 0.25 degree, 75 ms exposure per frame, tube current = 0.21 mA, and tube voltage = 50 kV. Images were reconstructed using MILabs Reconstruction software 11.00. The SPECT reconstruction was performed using an SROSEM algorithm with 9 iterations and 128 subsets, the energy window was centred at 140 keV +/- 20% and the voxel size was set to 200 μm isotropic. CT scans were reconstructed with a voxel size of 80 μm. SPECT images were co-registered to CT images and attenuation corrected. Region of interest analysis of the co-registered images was performed using VivoQuant image analysis software (inviCRO LLC 2021patch1hf1).

### Tissue collection and histological analysis

**For HE analysis and IHC**, mice were culled by cervical dislocation and lungs inflated with formalin. Lungs, kidneys, liver, spleen and brain were removed and incubated overnight in formalin at room temperature. Formalin was then replaced with 70% ethanol and samples stored at 4°C. The individual lung lobes were separated and positioned in the embedding cassette. Organs were embedded in paraffin and sections cut for H&E staining and other markers as appropriate.

Slides were scanned using a high-throughput scanner (Pannoramic 250 High Throughput Scanner, 3DHISTECH, Budapest, Hungary). Analysis of metastasis was undertaken using CaseViewer software (3DHISTECH). Analysis of other immunostainings was performed with QuPath image analysis software.

**For immunofluorescence analysis**, mice were culled by cervical dislocation and primary tumours and lungs inflated. Dissected tumours were fixed in 4%PFA+4%Sucrose in PBS solution for 1h at 4 °C with rotation then transferred to a 15% Sucrose in PBS solution overnight. The next day fixed tumours were imbedded in OCT and frozen at -80 degrees. 10 mm sections were cut.

For immunofluorescence, sections were dried for 30 min at RT, rehydrated in PBS, permeabilised with 0.5% Triton X-100, and blocked for 1 h in PBS containing 5% donkey serum, 0.2% BSA, 0.3% Triton X-100, and 0.05% NaN3. Sections were incubated overnight at 4°C with rabbit anti-Pdgfrβ (3169S, Cell Signaling, 1:200) and rat anti-Pecam (550274, BD Pharmingen, 1:100), or rat anti-endomucin antibody (SC-65495, Santa Cruz, 1:100) and rabbit anti-pgH2AX antibody (2577S, Cell Signaling, 1:100) followed by Alexa Fluor 488 anti-rabbit (A21206, Invitrogen, 1:300) and Alexa Fluor 594 anti-rat (SA5–10028, Thermo Fisher Scientific, 1:300) secondary antibodies for 1 h at RT. Sections were washed in PBS and mounted with ProLong Gold antifade reagent containing DAPI (Life Technologies).

Slides were either scanned using a high-throughput fluorescent scanner with a 20x objective and a sCMOS monochrome camera (NanoZoomer S60- Hamamatsu) or imaged in a Confocal Spinning disk microscope with a 20x Objective and sCMOS confocal camera (Nikon).

### Lung pre-metastatic endothelial cell (EC) FACS sorting and bulk RNA-sequencing

#### Lung processing and FACS sorting-

Pre-metastatic lung endothelial cells (ECs) were isolated from mouse lungs by enzymatic dissociation followed by negative depletion and fluorescence-activated cell sorting (FACS), adapted from Schereth et al.^40^. Briefly, lungs were excised under sterile conditions, rinsed in 70% ethanol for 2–3 s, transferred to MLEC medium, finely minced, and digested in collagenase type I (17100–017, GIBCO) prepared in PBS containing Ca^2^/Mg^2^ for 45 min at 37°C with 10 μg/ml DNase I (Sigma 10104159001). The digested tissue was mechanically dissociated by repeated passage through 19G and 21.5G needles, filtered through a 70 μm strainer, and centrifuged at 1,200 rpm for 3–5 min. Cell pellets were first incubated for 30 min at 4°C in FACS buffer (PBS + 5% heat-inactivated FBS) with antibodies against podoplanin/PDPN (53–5381, eBioscience, 1:100), LYVE1 (53–0443, eBioscience, 1:250), CD45/PTPRC (553080, BD Biosciences, 1:400) and Ter119/LY76 (561032, BD Biosciences, 1:200) for negative selection, followed by incubation with Dynabeads magnetic beads (11035, Life Technologies) for 30 min at 4°C on a rotator. After magnetic depletion, the supernatant was collected, washed, and incubated for 30 min at 4°C with antibodies against CD31 (551262, BD Biosciences, 1:100) and CD34 (48–0341, eBioscience, 1:50) for positive selection. Cells were then resuspended in FACS buffer containing propidium iodide (PI) (00–6990-50, eBiosciences, 1:2000), passed through a 40 μm strainer (352340, BD Falcon) or FACS tubes with cell-strainer caps (352235, BD), and kept on ice until sorting. Live endothelial cells were defined and sorted as PTPRC-LYVE1-LY76-PDPN-PI-CD31+CD34+ cells, directly into RLT lysis buffer (350–500 μl). A FITC-CD31-CD34-fraction was also collected for downstream validation of sort purity by qPCR.

#### mRNA sequencing-

RNA was extracted from sorted ECs using an RNA extraction microkit (Qiagen 74004), RNA amplified with SMART-Seq (V4 Ultra Low Input RNA kit for Sequencing Cat No. 634893, Takara Bio). Library preparation was carried out with polyA capture and sequencing performed on NovaSeq600 yielding on average ~30 million reads per sample, with 150bp paired-end (PE) reads, strand specific.

FASTQC was performed and quality trimming of the fastq files was applied using trimgalore v0.6.5. Alignment to the reference genome GRCm38 (mm10) was performed using STAR 2.7.0f alignment with 2-pass procedure^41^. Counting of reads was performed with RSEM using the Ensembl annotation GRCm38.102. One sample (S_15) was excluded due to poor quality. Only genes that achieved at least 10 counts in at least 4 samples each were kept for further analyses. This led to 16,163 filtered genes in total, 13,662 of which were protein coding genes. Downstream differential gene expression analysis was performed with R package DESeq2^42^.

### FACS analysis of pre-metastatic lung of P16-FDR model and lung experimental metastasis

Lungs were harvested into cold DPBS (1× DPBS prepared from 10× stock in ultra-pure water and sterile-filtered) and processed immediately. On an ice-cold Petri dish, the trachea, heart, and connective tissue were removed to isolate the five lung lobes. Lobes were transferred to a 2 mL tube, finely minced, and enzymatically digested in 1.5–2 mL per lung of freshly prepared sterile digestion buffer (HBSS containing Liberase TM and Liberase TH, each at 15.2 μL/mL, and DNase I at 5 μL/mL). Digests were incubated at 37°C with shaking (1200 rpm) for 30 min, then passed through a 70 μm strainer into a 50 mL tube and gently dissociated mechanically. Strainers were rinsed with quenching medium (DMEM containing +10% FBS) to maximise recovery, and samples were centrifuged at 300 × g for 10 min at 4°C. Pellets were resuspended in 1× red blood cell lysis buffer (Miltenyi Biotec, 130–094-183), immediately passed through a 40 μm strainer, and incubated for 3 min at room temperature. Cells were then washed, centrifuged again (300 × g, 10 min), and resuspended in PBS for downstream staining and flow cytometry analysis.

Single-cell suspensions were stained in 100 μL per sample. Unless otherwise stated, all steps were performed protected from light, and incubations were carried out on ice or at 4°C. Cells were first incubated with Fc receptor blocking reagent (BD, 553142, 1:200) together with DAPI (final 1:15,000 in PBS) for 20 min in the dark. Cells were then washed twice with FACS buffer (PBS, 2 mM EDTA, 2% FBS) by centrifugation (2000 rpm, 2 min, 4°C) and resuspended in 100 μL of extracellular antibody mastermix prepared in Brilliant Stain Buffer (BD, 566349). Surface staining was performed for 30 min on ice, followed by two washes in FACS buffer. Cells were subsequently fixed and permeabilised using 1× Fix/Perm solution (00–8222-49, Invitrogen, prepared 1:4 from concentrate) for ≥30 min, washed in 1× permeabilisation buffer (prepared as a 1:10 dilution in H_2_O), and washed an additional three times in FACS buffer. Finally, cells were resuspended in ≥200 μL FACS buffer, filtered through a 40 μm strainer into flow cytometry tubes, and stored at 4°C protected from light until acquisition.

For p16-FDR experiments, lung single-cell suspensions were stained with a Lineage cocktail comprising anti-CD45-FITC (BD Biosciences, 553080, 1:400), anti-LYVE1-FITC (53–0443, eBioscience, 1:250), and anti-Podoplanin-FITC (53–5381, eBioscience, 1:100), together with anti-CD31-APC (551262, BD, 1:100). DAPI was used to exclude non-viable cells. Cells were gated sequentially as live DAPI^-^, Lin^-^, and CD31^+^, and p16^+^ endothelial cells were identified by endogenous mCherry signal within the live Lin^-^ CD31^+^ gate.

For IV experimental metastasis experiments, lung single-cell suspensions were stained with CD45 (103149, Biolegend, 1:400), CD54 (116147, Biolegend, 1:50) and CD31 (551262, BD, 1:100). To assess tumour-cell homing, seeding, and colonisation, intravenously injected labelled LLC cells were identified as CellTrace^+^ CD45^-^ events and expressed as a percentage of live cells. To evaluate endothelial activation, lung endothelial cells were defined as CD45^-^ CD31^+^ cells, and the proportion of ICAM1^+^ cells was determined within CD45^-^ CD31^+^ gate.

### *In vitro* experiments

ECs (HPMECs and HUVECs) (see culture conditions above) were seeded at least 24 hours prior to start of treatment. Control as media and DMSO (5uM matched to FAKi), cisplatin (2.5ug/ml), FAKi (5uM PF 573228, #3239 TOCRIS), and combined cisplatin + FAKi were used. For the 5 days experiments, treatments were replenished with fresh media every 2–3 days.

#### B-Galactosidase and Cristal violet staining-

HPMECs were seeded in a 24 well plate and treated accordingly for 5 days and 5 days plus 3 without treatment. At the end point cells were stained with the senescence kit (ab65351, Abcam) or Cristal violet solution (V5265, Sigma-Aldrich) and imaged using a brightfield microscope (Olympus CKX41).

#### Immunocytochemistry and PLA-

Cells were plated in pre-coated 4 well chamber slides (Lab-Tek II Chamber Slide- 154524, ThermoFisher Scientific) and allowed to attach overnight, before treatment began. At the end of the corresponding time point, cells were fixed with 4% paraformaldehyde (PFA) for 15 min at room temperature; washed in PBS three times; and permeabilized in 0.2% Tx-100 in PBS for 10 min at room temperature; blocked with 0.1% BSA/0.2% Triton X-100 for 10 min at room temperature; washed three times in PBS; incubated for 1 h at room temperature with anti-p16 (ab189034, Abcam, 1:100), anti-pp53 (82530, Cell Signaling, 1:500), anti-FAK (610088, BD 1:100), anti-pgH2Ax (CS9718, Cell signalling 1:200) or anti-pATM (ab81292, Abcam 1:200); washed three times in PBS; incubated with Alexa-488- anti-rabbit (A32766TR, Invitrogen, 1:300) and/or alexa-546 anti-mouse (A10036, Invitrogen, 1:300) in PBS; washed three times in PBS; and, finally, mounted in Prolong Gold with DAPI. Duolink PLA in situ red starter mouse/rabbit kit was used according to manufacturer instructions (Sigma Aldrich, DUO92101). Images were acquired using a Confocal Spinning disk microscope with a 40x Objective and sCMOS confocal camera (Nikon).

For single time point ICC analysis (p-p53 and p16) nuclear fluorescence intensity was quantified in FIJI/ImageJ by segmenting nuclei in the DAPI channel using thresholding, despeckling, and watershed-based separation, followed by *Analyze Particles* to generate nuclear ROIs, which were then overlaid onto the relevant fluorescence channel for intensity measurement. Size thresholds were optimised empirically for each image set (typically a minimum particle size of 50 pixels for 40× images), with edge objects excluded where appropriate.

For all ICC analysis of several time points (Nuclear FAK, pgH2Ax and PLA) CellProfiler (Broad Institute) was used for automated nuclear segmentation and intensity quantification. Nuclei were identified from the nuclear channel by intensity-based thresholding with size filtering and declumping as needed. For each segmented nucleus, CellProfiler measured fluorescence intensity features for (including **Mean Intensity)**pgH2AX and FAK, and number of nuclear puncta for PLA.

#### Cleaved Caspase 3/7 assay-

HPMECs were stained with 10 mM Caspase-3/7 detection reagent (C10423, ThermoFisher Scientific) and plated in 96 well optical-bottom microplates (165305, ThermoFisher Scientific). Next day, treatment was added with the respective drugs as mentioned above and plate imaged using an Incucyte S3 IC52090 with a 20x objective over the course of 5 days. Positive cells (object counts) were quantified per well using detection threshold of green signal.

#### Secretome analysis-

HPMEC were treated for 5 days with the respective drugs as mentioned above. Collection of medium and removal of BSA was undertaken using an Albumin Depletion and Low Abundance Protein Enrichment Kit (AlbuVoid^™^, Caltag Medsystems Ltd, Buckingham, UK). Samples were quantified using a BCA assay; 50 μg were diluted in elution buffer (AlbuVoid^™^) to a final volume of 50 μl, then 50 μl of 4 M urea in 20 mM HEPES (pH 8.0) were added to obtain a protein suspension with 2 M urea. Sample volume was increased to 1 ml with 2 M urea in 20 mM HEPES (pH 8.0), 80 μl of conditioned trypsin beads [50 % slurry of TLCK-trypsin conditioned with 3 washes of 20 mM HEPES (pH 8.0)] were added and samples were incubated overnight at 37 °C with agitation. Trypsin beads were removed by centrifugation at 2,000 × *g* for 5 min at 5 °C and samples were desalted with Carbon C18 Top tips as indicated for proteomics experiments. Proteins present in the secretome pellets were identified and quantified following the procedure described for proteomics analysis (see bellow). A sample of serum supplemented, MV medium that was not exposed to ECs was subjected to BSA removal, processed for MS analysis and used as an internal control for medium proteins not secreted by ECs.

Samples were run in a randomized manner by shuffling samples before loading in a LC-MS/MS system. The LC system (Dionex UltiMate 3000 RSLC, Thermo-Fisher Scientific Inc) used mobile phases A (3 ACN: 0.1 % FA) and B (100 % ACN; 0.1 % FA). Peptides were trapped in a μ-pre-column (160454; Thermo Fisher Scientific Inc) and separated in an analytical column (EASY-SPRAY RSLC C18 2 μM, 50 CM X 75 μM, #ES903, Thermo-Fisher Scientific Inc). The following parameters were used: 3 % to 23 % B gradient for 120 min and a flow rate of 0.25 μl/min. As they eluted from the nano-LC system, peptides were infused into the online connected Q-Exactive Plus system via an Easy Spray Source. The instrument operated a 2.1 s duty cycle consisting in a full scan survey spectra (375–1,500 m/z) with a 70,000 FWHM resolution followed by, a data-dependent acquisition process in which the 15 most intense ions were selected for HCD (higher energy collisional dissociation) and MS/MS scanning (200–2,000 m/z) with a resolution of 17,500 FWHM. A 30 s dynamic exclusion period was enabled with an exclusion list with 10 ppm mass error window. Overall duty cycle generated chromatographic peaks of approximately 30 s at the base, which allowed the construction of extracted ion chromatograms (XICs) with at least 10 data points.

Mascot Daemon 2.6.0 (http://www.matrixscience.com/mascot_support_v2_6.html) automated peptide identification from MS data. Mascot Distiller v2.7.1.0 generated Peak list files (MGFs) from RAW data. Mascot search engine (v2.5) matched data stored in MGF files to peptides. Searches were performed against the SwissProt Database (uniprot_sprot_2014_08.fasta) with a FDR of ~1 % and the following parameters: 2 trypsin missed cleavages, mass tolerance of ±10 ppm for the MS scans and ± 25 mmu for the MS/MS scans, carbamidomethyl Cys as a fixed modification, PyroGlu on N-terminal Gln and oxidation of Met as variable modifications. The in-house developed Pescal software was used for label-free peptide quantification as previously indicated^43,44^, XICs for all the identified peptides across all samples were constructed with ±7 ppm and ±2 min mass and retention time windows, respectively. Peak areas from all XICs were calculated. Missed datapoints were given an intensity value equal to the minimal value obtained in the assessed sample divided by 10. Intensity values for each peptide were normalized to total sample intensity. Protein amount was inferred using the quantitative data for all peptides identified in the same protein.

All secreted proteins considered in the analysis presented an average amount across samples higher than in the reference sample not exposed to cells. Unpaired two tailed Student’s t-test was used to determine statistical differences in protein abundance between conditions and differences were considered significant when p > 0.05. Heatmaps and other visualization plots were constructed in R (v3.6.1https://www.r-project.org/) within the RStudio (v1.1.463 https://www.rstudio.com/) environment using the packages “ComplexHeatmap”( https://bioconductor.org/packages/release/bioc/html/ComplexHeatmap.html) and “ggplot2” (https://cran.r-project.org/web/packages/ggplot2/index.html) , respectively.

#### Adhesion assays-

Endothelial cells (HPMECs or HUVECs) were seeded in 4-well chamber slides and treated the following day in MV medium with control, cisplatin, FAK inhibitor, or cisplatin plus FAK inhibitor conditions for 5 days. At the end of the treatment period, ECs and tumour cells were labelled with CellTracker Red CMTPX (C34565, ThermoFisher Scientific) and CellTracker Green CMFDA (C7025, ThermoFisher Scientific) respectively, by incubation in OptiMEM containing dye (1:1000) for 40 min at 37°C, followed by quenching in full medium for 10 min. Tumour cells were then detached, resuspended in MV medium, and added to the endothelial monolayers for 1 h 30 min. Non-adherent cells were removed by a single careful PBS wash, after which cells were fixed in 4% paraformaldehyde (PFA). Images were acquired using a Confocal Spinning disk microscope with a 20x Objective and sCMOS confocal camera (Nikon).

#### Western blotting-

HUVECs were treated with cisplatin for 2 and 16h and 1-hour prior treatment of FAK inhibitor. Protein was extracted with Pierce^™^ RIPA buffer (1x) (#89900 Thermo scientific) with phosphatase (524625, Merck Millipore, 1:100) and protease inhibitors (539131, Merck Millipore, 1:100). Protein concentration was determined with BCA Protein Assay Kit (5000116, BIO-RAD) and samples were diluted in Pierce^™^ RIPA buffer (1x) (#89900 Thermo scientific) with phosphatase and protease inhibitors (1:100). BMER (2-mercaptoethanol) (M3148, Sigma Aldrich) was added in part 1:25 to NuPAGE^™^ LDS sample buffer (4x) (#11549166 Invitrogen) per sample. Proteins were separated by SDS-PAGE on 3–8% Tris-Acetate gels and transferred to PVDF membranes. Blots were separated into protein molecular weight and blocked with 5% milk in Tris-buffered saline with 0.1% Tween-20 (TBS-T) (P2287, Sigma Aldrich) for 1 hour at room temperature (RT). Primary antibodies anti-ATM (pS1981) (ab36810, Abcam, 1:2000), pFAK-Y397 (3283, CS, 1:250), anti-FAK (610088, BD, 1:200) and HSC70 (sc7298, Santa Cruz Biotechnology, 1:200) were diluted in 4% bovine serum albumin (BSA) (A9418, Sigma Aldrich) in TBS-T for all phospho-proteins and 5% milk TBS-T for non, then incubated over night at 4°C. Blots were washed for 30 minutes (6× 5mins) before the addition of secondary Horseradish peroxidase (HRP) conjugated sheep anti-mouse (515–035-003, Jackson ImmunoResearch Labs, 1:10000) or goat anti-rabbit, antibodies (31460, ThermoFisher Scientific, 1:10000), for 1 hour at RT. Dilutions followed the same criteria as primary antibodies. Blots were washed for another 30 minutes before exposure to Immobilon Classico Western HRP substrate solution (#WBLUC0500 Millipore). Western blot bands were quantified using Fiji^™^ to remove background noise and normalised against housekeeper proteins. Blots that assessed multiple proteins were stripped with Re-Blot Plus Mild Solution (1x) (#2502 EMD Millipore) for 18 minutes and blocked with 5% milk before continuing with primary and secondary antibodies.

### Statistical analysis

Unless specifically stated, all statistical analysis were performed using Graph Pad Prism v10 (Irvine, CA, USA). Specific details on each experiment statistical analysis are provided in the corresponding figure legends.

## Results

### Loss of endothelial cell focal adhesion kinase or its kinase function protects against cisplatin induced lung metastasis

**Figure 1:**
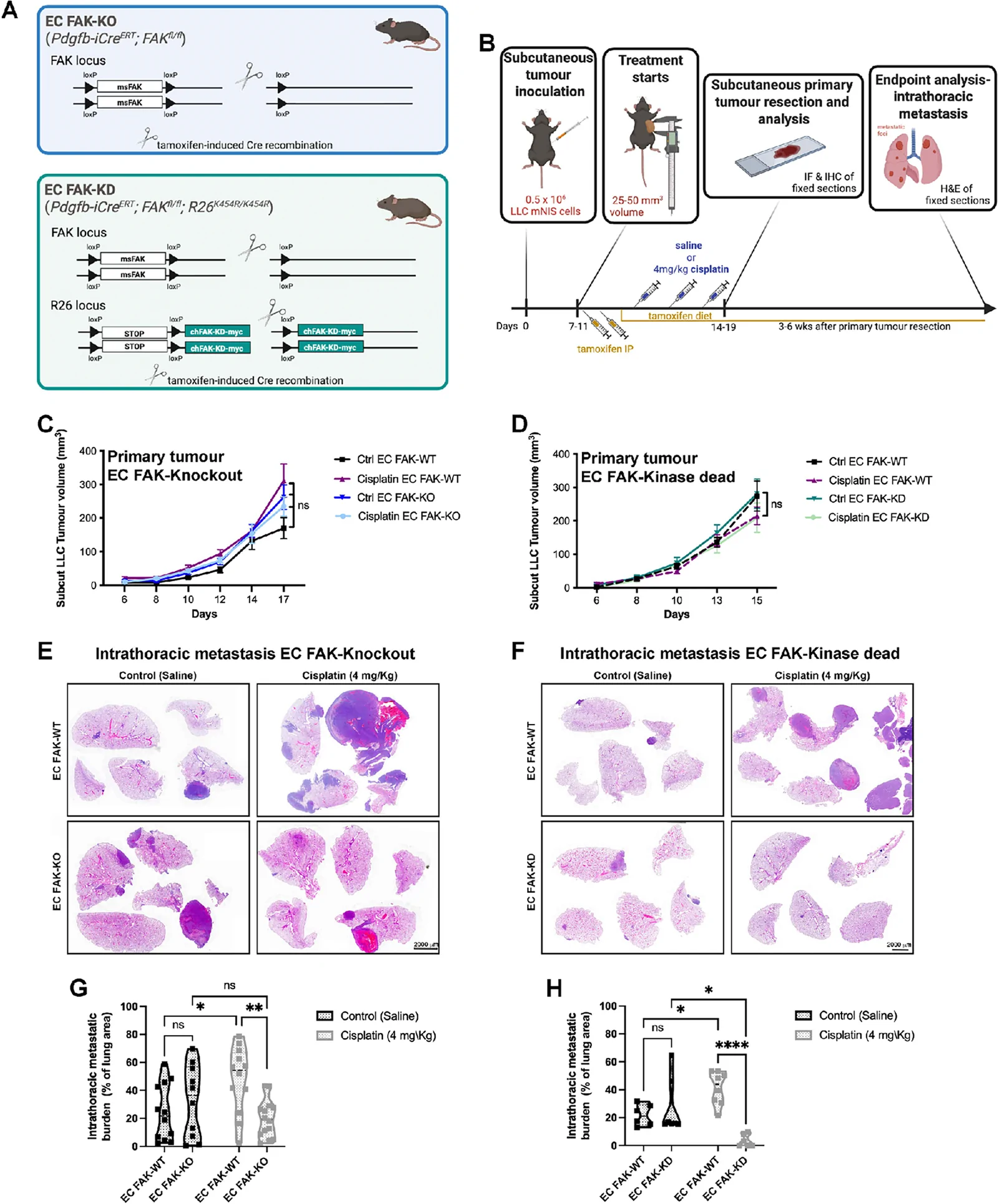
Loss of endothelial cell focal adhesion kinase or of its kinase function protects against cisplatin induced lung metastasis **(A**) Schematic representation of EC FAK-KO and EC FAK-KD mice (*Pdgfb-iCreERT; FAKfl/fl* and *Pdgfb-iCreERT; FAK*^*fl/fl*^*; R26*^*K454R/K454R*^ respectively). Tamoxifen treatment results in endothelial- FAK deleted mice (EC FAK-KO) and kinase dead mice (EC FAK-KD). Littermates with no *Pdgfb-iCreERT* (EC FAK-WT) mice, treated with tamoxifen, were used as controls. **(B)** Schematic representation of the experimental setup used to assess metastasis development in a neo-adjuvant setting- 0.5×10^6^ LLC mNIS-GFP cells (Lung carcinoma cells transduced with a mouse NIS- Sodium iodide symporter) were injected subcutaneously into the side belly of mice. Once tumours become established (25–50 mm^3^) specific EC FAK loss-of-function or kinase-dead are induced by tamoxifen administration. Mice then undergo three IP administrations of either Cisplatin (4 mg/Kg) or Saline (control group) every other day and imaged by SPECT to assess pre-metastatic status (no metastatic nodules present in secondary organs) the day before primary tumours resected. **(C)** and **(D)** Line graphs represent the mean LLC tumour volume (+/- sem) from day 6–17 post-injection. For panel C, n- 24 Ctrl EC FAK-WT mice; n-28 Ctrl EC FAK-KO mice; n-17 Cisplatin EC FAK-WT mice; n-20 Cisplatin EC FAK- KO mice. For panel D- n-10 Ctrl EC FAK-WT mice; n-10 Ctrl EC FAK-KD mice; n-10 Cisplatin EC FAK-WT mice; n-8 Cisplatin EC FAK-KD mice. **(E)** and **(F)** Representative H&E images of lungs with metastasis at end-point. **(G)** and **(H)** Violin plots denote Lung metastatic burden (% of lung area). For panels E and G, n- 12 Ctrl EC FAK-WT mice; n-10 Ctrl EC FAK-KO mice; n-12 Cisplatin EC FAK-WT mice; n-14 Cisplatin EC FAK- KO mice. For panel F and H, n-6 Ctrl EC FAK-WT mice; n-7 Ctrl EC FAK-KD mice; n-8 Cisplatin EC FAK-WT mice; n-6 Cisplatin EC FAK-KD mice. Each dot represents a mouse. A 2-way ANOVA test was used with a post-hoc Tukey’s multicomparison test. ns- not significant. * p<0.05; ** p<0.01; **** p<0.0001.

The involvement of platinum-based chemotherapy on lung cancer thoracic metastasis, and the effect of endothelial-cell FAK (EC FAK) loss or EC-FAK-kinase activity loss *in vivo* was investigated (Fig. 1A).

Lewis Lung carcinoma cells (LLCs) were implanted subcutaneously into the right flank of *Pdgfb-iCre*^*ERT+*^; *FAK*^*fl/fl* 33^ *or Pdgfb-iCre*^*ERT+*^; *FAK*^*fl/fl*^; *R26*^*K454R/K454R* 32^ mice, with *Pdgfb-iCre*^*ERT*-^; *FAK*^*fl/fl*^or *Pdgfb-iCre*^*ERT*-^; *FAK*^*fl/fl*^; *R26*^*K454R/K454R*^ mice used as respective controls. Once tumours were established (25–50 mm^3^) EC-FAK deletion or EC-FAK kinase-dead mutations, were induced by the administration of tamoxifen in *Pdgfb-iCre*^*ERT+*^; *FAK*^*fl/fl* 33^ *or Pdgfb-iCre*^*ERT+*^; *FAK*^*fl/fl*^; *R26*^*K454R/K454R* 32^ mice respectively. Tamoxifen treatment of *Pdgfb-iCre*^*ERT*-^; *FAK*^*fl/fl*^or *Pdgfb-iCre*^*ERT*-^; *FAK*^*fl/fl*^; *R26*^*K454R/K454R*^ acted as respective EC-FAK-WT controls. All mice were subsequently treated every other day with DNA-damaging cytotoxic chemotherapy, cisplatin (4 mg/Kg) or saline, as treatment controls. On the day after the last cisplatin administration, subcutaneous primary tumours were resected to allow the study of metastasis (Fig. 1B). EC FAK-knockout or EC-FAK kinase dead mutations had no effect on primary tumour growth with or without cisplatin treatment (Fig. 1C and D).

After primary tumour resections, most mice developed metastasis in the lungs, followed by mediastinal thoracic metastasis, while a very small percentage of mice developing liver, kidney or spleen metastasis (**Suppl. Figure 1A**). Cisplatin treatment increased intrathoracic metastatic burden in EC FAK-WT mice compared with saline treated EC FAK-WT mice, and this cisplatin-induced lung metastatic burden was decreased by the loss of endothelial cell FAK or endothelial cell-specific FAK kinase dead mutations in EC FAK-KO or EC FAK-KD mice (Fig. 1E-H). There was no correlation of lung metastasis with primary tumour relapse at the subcutaneous site (**Suppl. Figure 1B and C**) or the size of primary tumour at resection (**Suppl. Figure 1D and E**).

To validate these findings in a second model that generates spontaneous NSCLC, *Kras*^*FSF*-*G12D*^;*Trp53*^*fr/tfrt*^ mice^35^ were crossed with *Pdgfb-iCre*^*ERT*^; *FAK*^*fl/fl*^ to generate *Kras*^*FSF*-*G12D*^;*Trp53*^*fr/tfrt*^ ; *Pdgfb-iCre*^*ERT*^; *FAK*^*fl/fl*^ mice. In this model, through intralobular injection of Adenovirus-Flippase (Adeno-FlpO) directly in the left lung lobe, a primary tumour is generated and capable of metastasising to distant organs and counterlateral lung lobes, closely mimicking the clinical presentation and progression of lung cancer^39^. Following primary lung tumour establishment at approximately week 7 post-injection, KP EC-FAK deletion is induced by the administration of tamoxifen in KP *Pdgfb-iCre*^*ERT+*^; *FAK*^*fl/fl*^, as detailed above (**Suppl. Figure 2A**). Tamoxifen treatment of KP *Pdgfb-iCre*^*ERT*-^; *FAK*^*fl/fl*^ acted as respective KP EC-FAK-WT controls. All mice were then subsequently treated every week for 4 weeks with either cisplatin (4 mg/Kg) or saline, as treatment controls (**Suppl. Figure 2A**). Computed tomography (CT) imaging was used to track tumour growth from week 6 and every other 3 weeks from then on to assess response to treatment. Primary tumour volume was not significantly different between saline-treated KP EC FAK-WT and EC FAK-KO mice (**Suppl. Figure 2B and C**). In contrast, cisplatin-treated KP EC FAK-KO mice showed decreased tumour growth compared with respective cisplatin-treated KP EC FAK-WT mice (**Suppl. Figure 2B and C**). Importantly, all mice groups presented small lung metastasis in either left or right lung lobes, except for cisplatin treated KP EC FAK-KO mice, in which only less that 50% of mice had counterlateral right lung lobe metastasis (**Suppl. figure D and E**). Reflecting the different rates of primary tumour growth and metastatic progression, EC FAK loss by itself did not significantly affect overall survival compared with EC FAK-WT controls (**Suppl. Figure 2F**). However, following cisplatin treatment, KP EC FAK-KO mice showed improved survival compared with cisplatin-treated KP EC FAK-WT mice (**Suppl. Figure 2F**), indicating that endothelial cell FAK deletion alone does not alter disease progression in untreated KP tumours, but suggesting that it does protect against the pro-metastatic effects of chemotherapy.

Together, these data suggest that in the two lung models presented here, primary tumour growth is not affected by cisplatin treatment or EC-FAK loss or kinase activity. However, lung metastatic burden is significantly reduced after cisplatin treatment in mice lacking EC-FAK or EC-FAK-kinase activity compared with EC-FAK-WT controls.

### Endothelial FAK loss or kinase dead preserves primary tumour vessel density and pericyte coverage after cisplatin treatment

**Figure 2:**
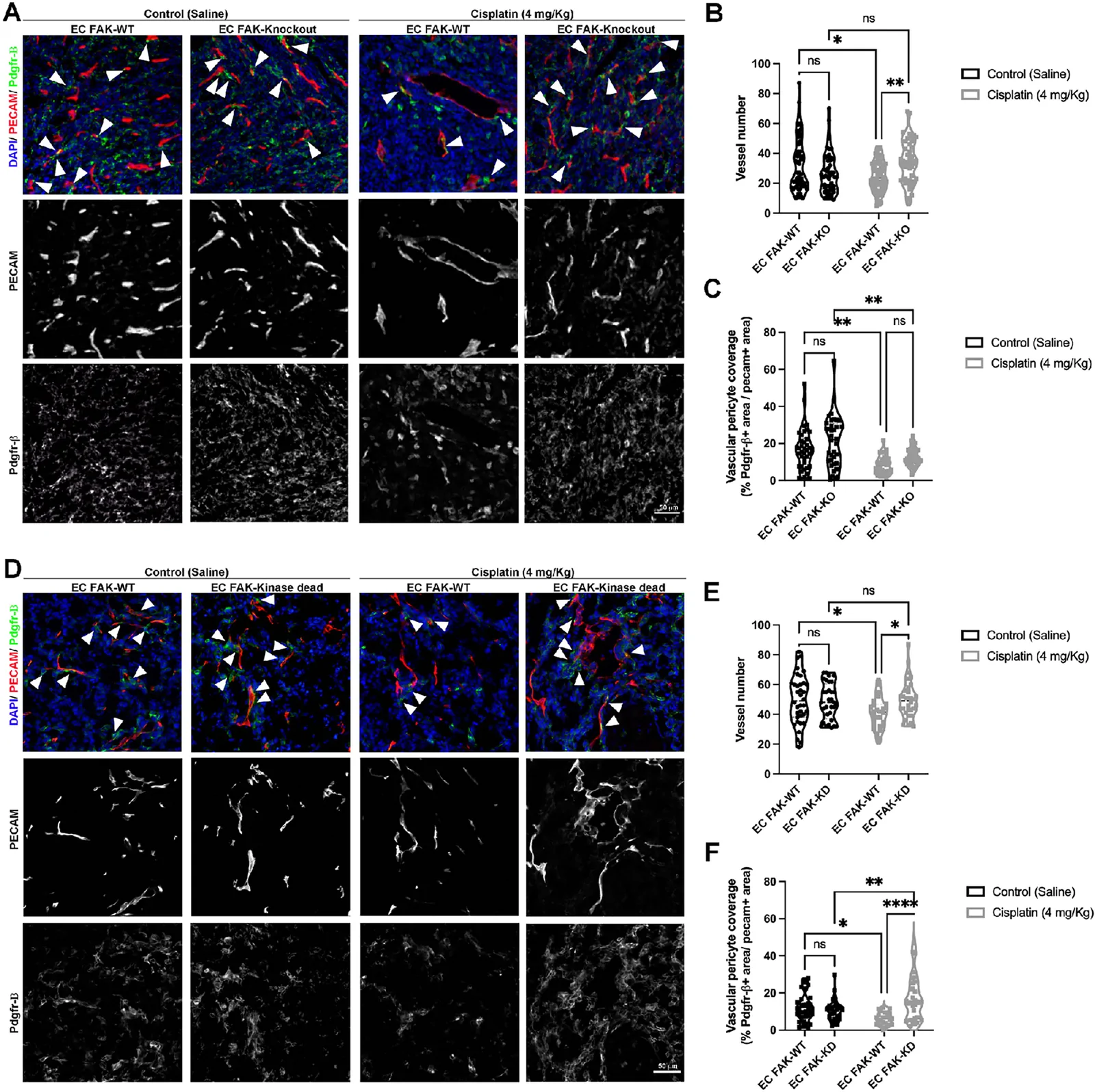
Endothelial FAK loss or a kinase dead version preserves primary tumour vessel density and function during cisplatin treatment **(A)** and **(D)** Representative immunofluorescence images of resected primary tumours stained for Pecam (red), Pdfgr-b (green) and DAPI (blue). White arrows indicate vessel coverage by pericytes (Pdgfr-b). **(B), (C), (E)** and **(F).** Violin plots denote individual measurements per field of Vessel number **(B)** and **(E)**, and Vascular pericyte coverage **(C)** and **(F)** in control (black) and cisplatin (grey). A 2-way ANOVA test was used with a post-hoc Tukey’s multi-comparison test. ns- not significant. * p<0.05; ** p<0.01; **** p<0.0001. A range of 3–20 fields p/mouse were analysed. For panels A-C, n-3 Ctrl EC FAK-WT mice; n-4 Ctrl EC FAK-KO mice; n-4 Cisplatin EC FAK-WT mice; n-5 Cisplatin EC FAK- KO mice. For panels D-F, n-9 Ctrl EC FAK-WT mice; n-8 Ctrl EC FAK-KD mice; n-7 Cisplatin EC FAK-WT mice; n-8 Cisplatin EC FAK-KD mice.

To investigate how EC FAK regulates LLC lung metastasis formation upon cisplatin treatment, we first analysed effects on the subcutaneous primary LLC tumour vasculature. Cisplatin treatment of EC FAK-WT mice reduced both the number of primary tumour blood vessels as well as pericyte coverage (measured by Pdgfr-β blood vessel coverage) indicating loss of vessel stability (Fig. 2A-F). Notably, after cisplatin treatment, the loss of EC-FAK or EC-FAK kinase dead mutation in EC FAK-KO and EC FAK-KD mice, respectively, rescued primary tumour blood vessel numbers compared with EC FAK-WT (Fig. 2A, B, D and E). Although, cisplatin treatment of EC FAK-KO mice retained the loss of pericyte coverage compared with cisplatin treated EC FAK-WT controls, pericyte coverage was rescued in primary tumours of EC FAK-KD mice compared with cisplatin treated EC FAK-WT control mice (Fig. 2A, C, D and F and **Suppl. Figure 3A-B**). Consistent with the rescue in vascular stability, cisplatin treatment of EC FAK-KO mice or KD mice increased blood vessel perfusion function measured in anti-mortem lectin perfusion experiments and histological analysis of the percentage of lectin-positive blood vessels (**Suppl. Figure 3A-D**). Altogether these data suggests that EC FAK and its kinase function partially protect against the vascular disruptive effects of cisplatin treatment in subcutaneous primary tumours possibly contributing to reduced lung metastasis formation in a neo-adjuvant setting.

### Cisplatin treatment elicits a FAK-dependent endothelial cell stress response in pre-metastatic lungs

Given that our results indicate that EC FAK loss, or its loss of kinase activity, can reduce metastatic burden to the lung, with associated changes in primary tumour vascular responses to cisplatin, we next examined if this correlated with changes in the pre-metastastic lung vascular endothelial cells. The same experimental setup was used as above (in Fig. 1B) but making use of LLC cells expressing mNIS (mouse sodium iodide symporter), which together with SPECT/CT (single-photon emission computed tomography*)*, a highly sensitive method for tracking metastatic dissemination, provided to be an ideal model for early metastasis detection^37,38^.

**Figure 3:**
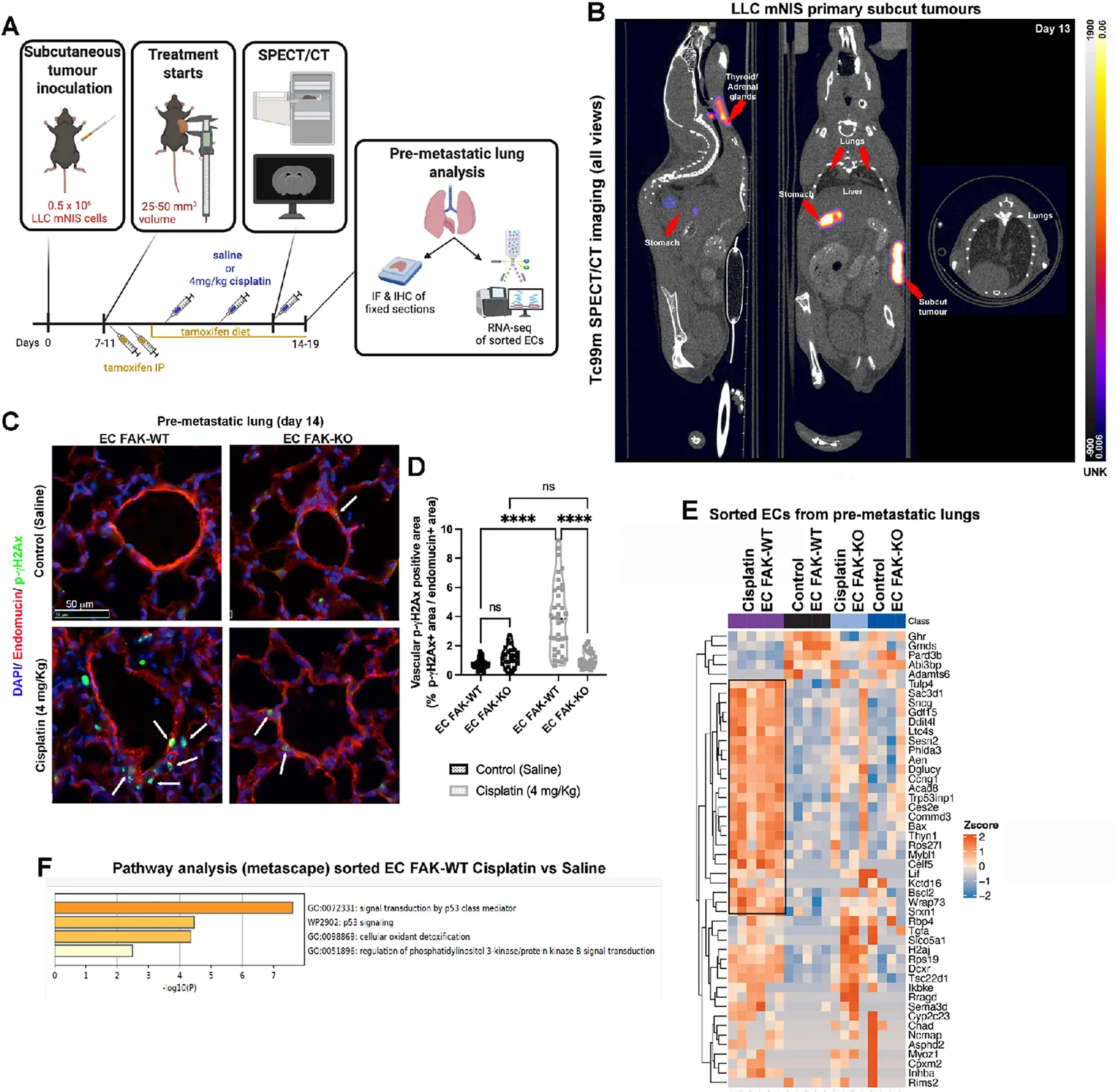
Cisplatin treatment elicits a FAK-dependent endothelial-cell stress response in the pre-metastatic lungs **(A)** Schematic representation of the experimental setup used to assess pre-metastatic lung niche in a neo-adjuvant setting- 0.5×10^6^ LLC mNIS-GFP cells (Lung carcinoma cells transduced with a mouse NIS- Sodium iodide symporter) were injected subcutaneously into the side belly of mice. Once tumours become established (25–50 mm^3^) specific EC FAK loss-of-function is induced by tamoxifen administration. Mice then undergo three IP administrations of either cisplatin (4 mg/Kg) or saline (control group) every other day and imaged by SPECT to assess pre-metastatic status (no metastatic nodules present in secondary organs) the day before lungs are collected. **(B)** Representative SPECT/CT image of mice administered with Tc99m (IV) that is up-taken by tumour cells (shown as primary tumour site) and by background physiological uptake in the stomach and thyroid. **(C)** Representative immunofluorescence image of resected primary tumours stained for Pecam (red), p-gH2AX (green) and DAPI (blue). White arrows indicate vascular DNA damage. **(D)** Violin plots denote Vascular p-gH2AX positive area per field. A 2-way ANOVA test was used with a post-hoc Tukey’s multi-comparison test. ns- not significant. * p<0.05; **** p<0.0001. A range of 7–12 fields p/mouse were analysed. n-3 Ctrl EC FAK-WT mice; n-3 Ctrl EC FAK-KO mice; n-4 Cisplatin EC FAK-WT mice; n-3 Cisplatin EC FAK-KO mice. **(E)** Heatmap of uniquely differentially expressed genes between cisplatin EC FAK-WT and control (saline) EC FAK-WT sample groups (Benjamini-Hochberg adjusted p≤0.05, generalised linear model). Black rectangle highlights a cluster of the uniquely upregulated genes with a homogeneous pattern of significantly higher expression in Cisplatin WT but not in Cisplatin KO versus their respective saline controls. n-5 Ctrl EC FAK-WT; n-4 Ctrl EC FAK-KO; n-4 Cisplatin EC FAK-WT; n-6 Cisplatin EC FAK-KO mice. **(F)** Metascape pathway analysis of the uniquely upregulated genes between cisplatin EC FAK-WT and control (saline) EC FAK-WT sample groups highlighted within the black rectangle. For panels E and F, n-5 Ctrl EC FAK-WT; n-4 Ctrl EC FAK-KO; n-4 Cisplatin EC FAK-WT; n-6 Cisplatin EC FAK-KO mice.

LLC mNIS GFP cells were injected subcutaneously into the right flank of *Pdgfb-iCre*^*ERT+*^; *FAK*^*fl/fl* 33^ and *Pdgfb-iCre*^*ERT*-^; *FAK*^*fl/fl*^; tumour growth was measured using callipers and once primary subcutaneous tumours became established (25–50mm^3^) all mice were treated with tamoxifen to induce EC FAK-KO in *Pdgfb-iCre*^*ERT+*^; *FAK*^*fl/fl*^, but not in *Pdgfb-iCre*^*ERT*-^; *FAK*^*fl/fl*^ *mice* ^33^ which acted as EC FAK-WT controls. Mice were then treated with cisplatin or saline (as treatment controls) (Fig. 3A). 24h after the final cisplatin treatment, radiolabelled Tc99m SPECT/CT imaging revealed no detectable radioactivity in the lungs regardless of clear radioactivity in the subcutaneous primary tumour (and expected background physiological uptake in bladder, stomach, thyroid and adrenal glands). This confirmed the absence of lung metastasis, allowing examination of the lung at this timepoint for pre-metastatic niche features (Fig. 3B). Immunofluorescence analysis of sections of the lungs collected at this time point revealed significantly increased DNA-damage in the pre-metastatic lung vasculature (p-γH2Ax+/ Endomucin+) in cisplatin treated WT mice compared with saline treated EC FAK-WT controls. In contrast, EC FAK loss in EC FAK-KO mice protected against cisplatin induced DNA damage significantly (Fig. 3C and D), suggesting a functional requirement for EC FAK in DNA-damage responses induced by cisplatin in the pre-metastatic niche vasculature.

To examine transcriptional changes in the pre-metastatic niche, vascular endothelial cells were sorted from the pre-metastatic lungs of another cohort of cisplatin treated or saline treated mice with EC FAK deletion and RNA sequencing performed (**Suppl. Figure 4A**). Comparing the differentially expressed genes between cisplatin and saline, 42 were uniquely upregulated in EC FAK-WT mice, whereas 147 were uniquely upregulated in EC FAK-KO mice. However, these 147 were also highly expressed in Cisplatin and Saline in WT mice (**Suppl. Figure 4D**), highlighting their potential negative regulation by EC FAK in Control conditions. The uniquely upregulated gene signature in cisplatin treated EC FAK WT mice, and abrogated in EC FAK KO mice, was characterised by robust induction of DNA damage– and p53-responsive genes, including *Trp53inp1*, *Ccng1*, *Phlda3*, *Bax*, *Thyn1* and *Rps27l*, together with stress-adaptive regulators *Sesn2* and *Ddit4l*, consistent with activation of a genotoxic and oxidative-stress response and suppression of mTOR signalling^45–47^ (Fig. 3E-F). The coordinated upregulation of *Trp53inp1*, *Phlda3*, *Sesn2*, *Ddit4l* and *Gdf15* is indicative of the emergence of a senescence endothelial phenotype, encompassing growth arrest, metabolic reprogramming and enhanced stress-response signalling^46–50^. Parallel induction of paracrine mediators, including *Gdf15* and *Lif*, and of the leukotriene biosynthetic enzyme *Ltc4s* implicates cytokine and lipid-mediator release as functional outputs of this state^49,51,52^. Conversely, comparing the differentially expressed genes between cisplatin and saline, 150 were uniquely downregulated in EC FAK-KO mice (**Suppl. Figure 4C and D**). Accordingly, endothelial FAK deficiency resulted in reduced expression of multiple p53-dependent checkpoint and DNA repair genes following cisplatin exposure, including the p53 targets^45,53^ *Cdkn1a*, *Mdm2*, *Phlda3* and *Bax*, as well as DNA repair regulators^54^ *Ercc1*, *Rad54b* and *Rad1 (***Suppl. Figure 4D and E***)*. In addition to reduced induction of DNA damage response genes, FAK-deficient endothelial cells exhibited increased expression of several negative regulators and modulators of DDR signalling, including the γH2AX phosphatase regulatory subunit *Ppp4r2*^55^, the p53 suppressor *Mdm4*^56^, and chromatin modifiers *Ehmt1* and *Pbrm1*^57^ *(***Suppl. Figure 4E).**

Collectively, these data indicate that cisplatin exposure elicits a FAK-dependent endothelial stress-response that integrates p53-dependent checkpoint and DNA-repair activation, oxidative-stress signalling, metabolic adaptation, senescence reprogramming and paracrine activation (Fig. 3E and F and **Suppl. Figure 4D and E**), consistent with endothelial priming of the pre-metastatic lung microenvironment.

### Cisplatin treatment triggers vascular endothelial-cell senescence, and loss-of FAK or FAK kinase inhibition promotes a senolytic effect

**Figure 4:**
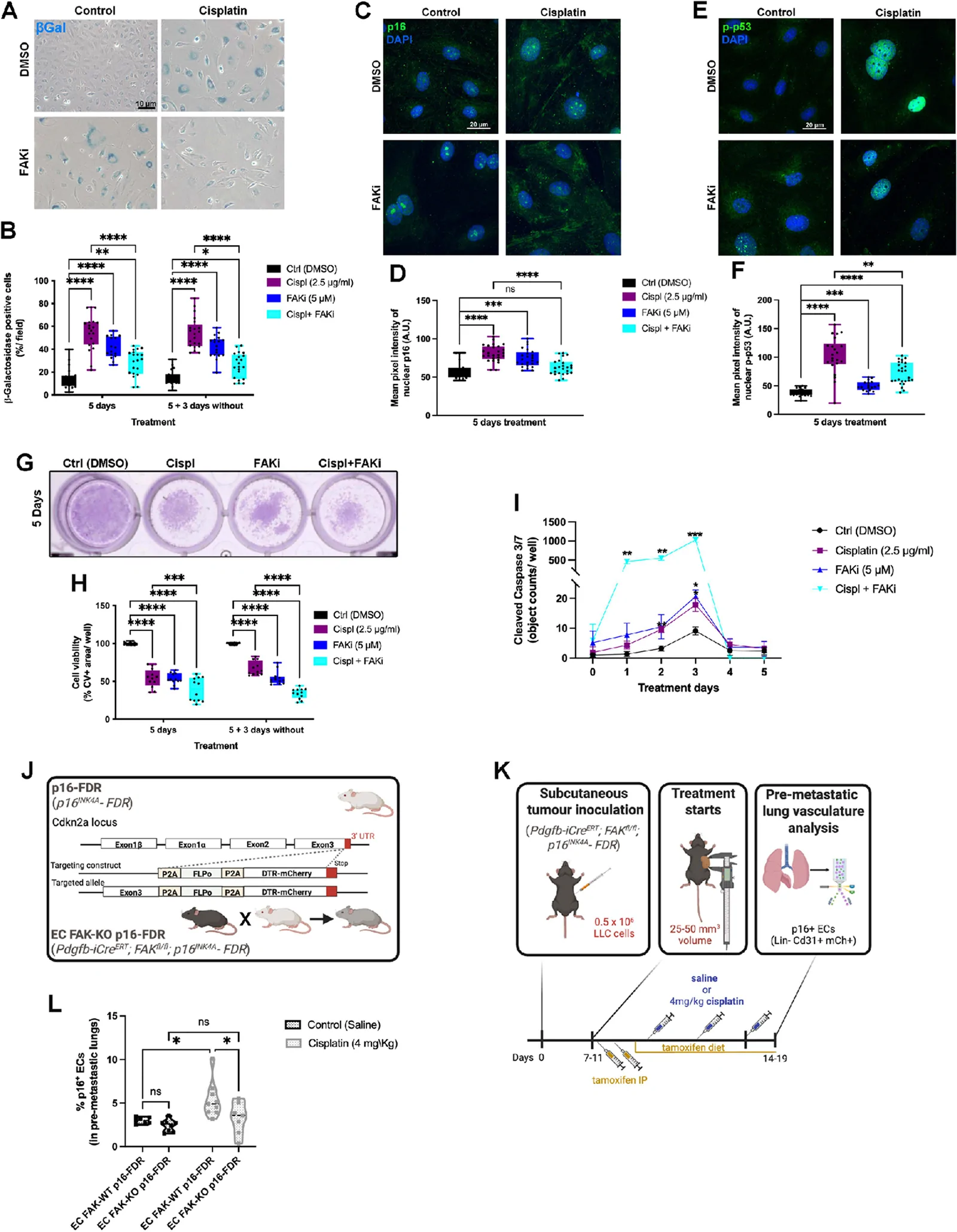
Cisplatin treatment triggers vascular endothelial-cell senescence, with loss-of FAK or its kinase inhibition promoting a senolytic effect HPMECs were treated for 5 days with Cisplatin, FAKi, or combination of both, or for 5 days plus 3 without the respective drugs. **(A)-(H)** Cells were fixed and stained for several senescence markers. **(A)** Representative brightfield images of b-Galactosidase staining, showing senescent cells marked in blue. (**B)** Bar graphs denote mean number of positive cells per field (in %) (+/- sem). A range of 16–21 fields p/condition were analysed across 2 biological replicates. A 2 way-ANOVA with Tukey’s multicomparison test was performed. **(C)** and **(E)**. Representative immunofluorescence images showing p16 (green) **(C)** or phospho-p53 **(E)**. (**D)** and **(F)** Distributions of per-field mean pixel nuclear intensities were visualised as boxplots with overlaid jittered points. A range of 16–27 fields p/condition were analysed across 2 biological replicates. A Brown-Forsythe and Welch ANOVA analyses with a Dunnett’s T3 multicomparison test was performed. **(G)** Representative image of Crystal violet staining of HPMECs in respective treated well plate. **(H)** Bar graphs denote mean well positive area (in %) (+/- sem). 3 wells p/condition were analysed across 2 biological replicates. A 2 way-ANOVA with Tukey’s multicomparison test was performed. **(I)** Cell event Caspase-3/7 green detection reagent was used to detect cells undergoing apoptosis. Green signal was captured using an incucyte S3. Line graph shows the mean value (+/-sem) of positive object counts/well, normalized to day 0. 5 wells p/condition were analysed across 2 biological replicates. A 2 way-repeated measures ANOVA with Dunnets’s multicomparison test was performed. **(J)** Schematic representation of a p16 reporter mouse line. EC FAK-KO strain (*Pdgfb-iCre*^*ERT*^*; FAK*^*fl/fl*^) was crossed with a reporter p16 strain (*p16*^*INK4A*^*-FDR*)^34^ to generate EC FAK-KO p16-FDR mice (*Pdgfb-iCre*^*ERT*^*; FAK*^*fl/fl*^*; p16*^*INK4A*^*-FDR)*. **(K)** Schematic representation of the experimental setup used to assess pre-metastatic lung niche in a neo-adjuvant setting- 0.5×10^6^ LLC mNIS-GFP cells (Lung carcinoma cells transduced with a mouse NIS- Sodium iodide symporter) were injected subcutaneously into the side belly of *Pdgfb-iCre*^*ERT*^*; FAK*^*fl/fl*^*; p16*^*INK4A*^*-FDR* mice. Once tumours become established (25–50 mm^3^) specific EC FAK loss-of-function is induced by tamoxifen administration. Mice then undergo three IP administrations of either cisplatin (4 mg/Kg) or saline (control group) every other day and lungs collected for FACS analysis the day after the last cisplatin administration. **(L)** Violin plot denotes percentage of Lin^-^ (Cd45, Lyve1, Podoplanin) Cd31^+^ p16mCherry^+^ ECs in pre-metastatic lungs. A 2-way ANOVA test was used with a post-hoc Tukey’s multicomparison test. Each dot represents one mouse. n-7 Ctrl EC FAK-WT p16-FDR mice; n-8 Ctrl EC FAK-KO p16-FDR mice; n-9 Cisplatin EC FAK-WT p16-FDR mice; n-7 Cisplatin EC FAK-KO p16-FDR mice. ns- not significant. * p<0.05; ** p<0.01; **** p<0.0001.

We then tested whether Cisplatin would induce a senescence phenotype in endothelial cells both *in vitro* and *in vivo*. Treatment of human pulmonary microvascular endothelial cells (HPMECs) with either Cisplatin or FAK kinase inhibitor (FAKi) (for 5 days) increased the accumulation of lysosomes and lysosomal hydrolase activity, as measured by β-galactosidase staining (Fig. 4A-B), as well as of other senescence markers, such as p16 (Fig. 4C-D) and p-p53 (Fig. 4E-F). Importantly, withdrawal of the drugs for an extra 3 days did not reverse the increase in β-galactosidase positive cells, reinforcing that the effect of cisplatin and FAKi on HPMECs is a permanent senescent state (Fig. 4A-B). Interestingly, the combination of cisplatin with inhibition of FAK kinase activity decreased these markers of senescence, suggesting that combined treatment either inhibits senescence or has a potential senolytic effect.

Indeed, and even though single treatment with either cisplatin or FAKi reduced cell viability, the combined treatment produced a more pronounced reduction in cell viability, that was also not reversed by an extra 3 day drug withdrawal (Fig. 4G-H). Furthermore, using cleaved caspase 3/7 assay throughout the 5 days treatment, revealed that combined treatment led to higher levels of apoptotic death in ECs, when compared with single treatments or control conditions (Fig. 4I).

To assess if cisplatin treatment induces senescence *in vivo* in the pre-metastatic lung vasculature, and if this is regulated by FAK, we crossed *Pdgfb-iCre*^*ERT+*^; *FAK*^*fl/fl*^ mice with a p16-reporter strain *p16*^*INK4A*^*-FDR* ^34^ to generate *Pdgfb-iCre*^*ERT+*^; *FAK*^*fl/fl*^; *p16*^*INK4A*^*-FDR mice* (Fig. 4J). Making use of the same previous experimental setup, LLC cells were injected subcutaneously into the right flank of *Pdgfb-iCre*^*ERT+*^; *FAK*^*fl/fl*^; *p16*^*INK4A*^*-FDR* and *Pdgfb-iCre*^*ERT*-^; *FAK*^*fl/fl*^; *p16*^*INK4A*^*-FDR;* tumour growth was measured using calipers and once primary subcutaneous tumours became established (25–50mm^3^) all mice were treated with tamoxifen to induce EC-FAK KO in *Pdgfb-iCre*^*ERT+*^; *FAK*^*fl/fl*^ ; *p16*^*INK4A*^*-FDR*, but not in *Pdgfb-iCre*^*ERT*-^; *FAK*^*fl/fl*^; *p16*^*INK4A*^*-FDR mice*, which acted as EC-FAK-WT p16-FDR controls (Fig. 4K). Mice were then treated with cisplatin or saline (as treatment controls) and 24h after the last cisplatin treatment, pre-metastatic lungs were collected, processed and analysed by FACS to assess the induction of p16 expression (measured by Lin-Cd31 + mCherry+) in lung vascular ECs (Fig. 4K). Cisplatin increased expression of p16 in pre-metastatic lung vascular endothelial cells of EC FAK-WT p16-FDR mice compared with saline treated EC FAK-WT p16-FDR mice. In contrast, cisplatin treatment of EC FAK-KO p16-FDR mice showed no effect on EC p16 expression (Fig. 4L). These data suggest that Cisplatin induces vascular endothelial cell senescence in pre-metastatic lungs, and that this combined with the loss of EC FAK, or FAK kinase inhibition, promotes a senolytic effect that might contribute to decreased lung metastasis formation.

### Cisplatin induces a FAK-dependent endothelial cell secretory programme associated with increased adhesion of tumour cells and lung metastasis, independent of the presence of a primary tumour

**Figure 5:**
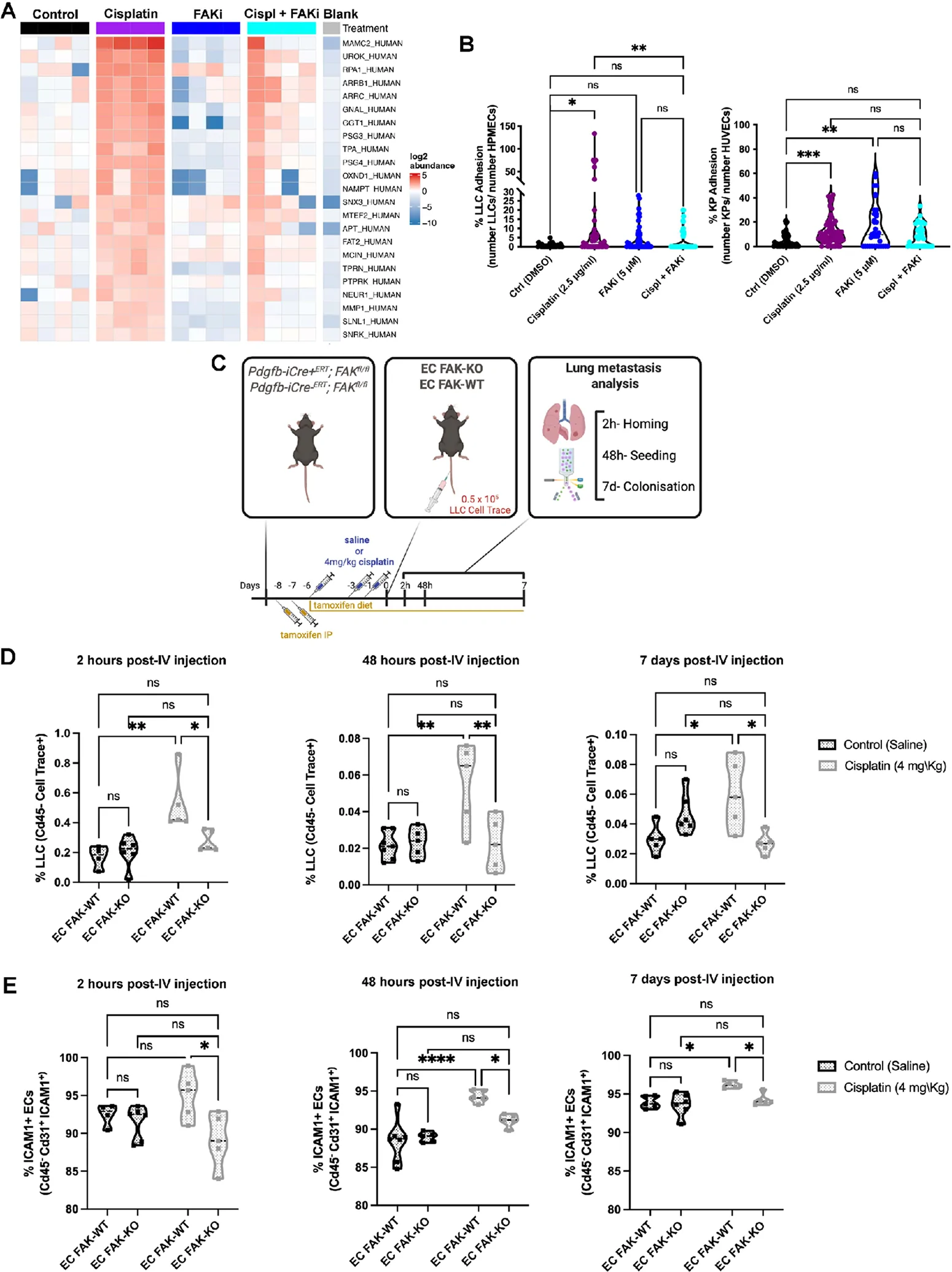
Cisplatin induces a FAK-dependent endothelial secretome in vascular endothelial cells associated with increased adhesion of tumour cells and lung metastasis, independently of the presence of a primary tumour **(A**) HPMECs were treated for 5 days with either cisplatin, FAK inhibitor or combination, after which media was collected for secretomics analysis. Heatmap of significantly differently secreted proteins. n- 4 biological replicates p/ condition. **(B)** Violin plots denote the number of TCs per field (in %) that adhere to ECs, previously treated for 5 days (and normalized to the number of ECs present). For LLC/HPMEC adhesion, a range of 7–20 fields p/ condition were analysed across 3 biological replicates. For KP/HUVEC adhesion, a range of 7–26 fields p/condition were analysed across 3 biological replicates. A Kruskal-Wallis non-parametric analysis was performed with a Dunn’s multiple comparison test. **(C)** Schematic representation of experimental metastasis with neo-adjuvant Cisplatin treatment in EC FAK-WT and KO mice. **(D)** Violin plots denote percentage of live CD45^-^LLC^+^ cells in lungs after 2h, 48h and 7d of IV injection. **(E)** Violin plots denote percentage of live CD45-CD31+ ICAM1+ lung ECs after 2h, 48h and 7days after IV injection. Each dot represents one mouse. For 2 hours, n-4 control EC FAK-WT, n-6 control EC FAK-KO, n-5 cisplatin EC FAK-WT, and n-5 cisplatin EC FAK-KO. For 48 hours, n-7 control EC FAK-WT, n-5 control EC FAK-KO, n-5 cisplatin EC FAK-WT and n- 5 cisplatin EC FAK-KO. For 7 days, n-5 control EC FAK-WT, n-6 control EC FAK-KO, n-5 cisplatin EC FAK-WT and n- 5 cisplatin EC FAK-KO. A 2-Way ANOVA with Tukey’s multiple comparison test was performed. ns- not significant. * p<0.05; ** p<0.01; **** p<0.0001.

Since one of the features of senescent cells is a hypersecretory state, known as senescence, associated secretory phenotype (SASP), that exerts a multitude of paracrine effects to their immediate microenvironment^58^, we investigated the effect of cisplatin and EC FAK on the secretome. Mass spectrometry profiling of human pulmonary endothelial cells identified a cisplatin-induced, FAK-dependent secretory signature associated with metastatic permissiveness (Fig. 5A). Cisplatin treatment robustly increased secretion of extracellular proteases, inflammatory mediators, and adhesion-regulating factors, and some established SASP factors^59^, all of which were consistently suppressed by pharmacological FAK inhibition. MAM domain-containing protein 2 (MAMC2), has been implicated in cell–cell and cell–matrix interactions, and emerging evidence suggests a role in regulating tumour–stromal crosstalk and epithelial–mesenchymal transition in colorectal cancer models^60^. Urokinase (UROK or uPA) and tissue plasminogen activator (TPA or tPA), key components of the plasminogen activation system that promote extracellular matrix degradation, endothelial barrier disruption and drive directed migration of tumour cells^61,62^. Matrix metalloprotease-1 (MMP1), linked to vascular permeability and tumor cell extravasation^63,64^. Nicotinamide Phosphoribosyltransferase (NAMPT), a chemotactic cytokine-like factor known to promote endothelial activation, upregulation of adhesion molecules and EC barrier function^65^, and a marker of chronic inflammation, therapy induced SASP and cancer progression^66,67^. Pregnancy-specific glycoproteins (PSG3/PSG4), immunomodulatory glycoproteins with established roles in angiogenesis and immune remodeling^68^. γ-glutamyltransferase 1 (GGT1), an enzyme associated with oxidative stress and endothelial dysfunction^69^ consistent with chemotherapy-induced vascular injury. Neuraminidase (NEUR1), a sialidase that modulates endothelial surface glycosylation and enhances cellular adhesiveness^70^. Receptor-type protein tyrosine phosphatase kappa (PTPRK), a junctional regulator, indicative of endothelial junction remodeling^71^. Finally, several intracellular proteins associated with DNA damage and cellular stress (including RPA1, SNX3, and SNRK), consistent with endothelial injury and/or vesicular release were all increasingly secreted upon cisplatin treatment, compared with non-treated control conditions (Fig. 5A).

Together, these changes in secreted factors suggest that cisplatin induces an endothelial secretory program that is abrogated by FAK inhibition, and these not only potentially increase vascular permeability, but also actively attract tumour cells, promoting their adhesiveness to the lung vasculature, potentially facilitating metastasis. To test this directly, we performed *in vitro* tumour cell adhesion assays on human ECs (HPMECs and HUVECs) pre-treated with cisplatin or FAKi alone or combination of both. Cisplatin treatment promoted increased adhesion of lung tumour cells (LLC and KP) to endothelial cells while combined treatment abrogated this effect (Fig. 5B and **Suppl. Figure 5A**).

To investigate if Cisplatin-induced senescent reprogramming in pre-metastatic lung vasculature was sufficient to promote metastasis in the absence of a primary tumour, LLC cells, labelled with cell tracker, were injected via the tail vein into cisplatin treated EC FAK-WT and KO mice (generated by tamoxifen administration in *Pdgfb-iCre*^*ERT*-^; *FAK*^*fl/fl*^ and *Pdgfb-iCre*^*ERT+*^; *FAK*^*fl/fl*^, *respectively* ) (Fig. 5C). We assessed tumour cells in lungs by FACS at 2h (homing), 48h (seeding) and 7d (colonisation) post IV injection. Cisplatin treatment in EC FAK-WT promoted increased number of tumour cells in the lungs in all the time points analysed and this effect was abrogated in EC FAK-KO mice (Fig. 5D). ICAM1 has been implicated in tumour cell adhesion to endothelial cells, transendothelial cell migration, and lung metastatic seeding^72,73,74^. Accordingly, cisplatin treatment in EC FAK-WT mice also promoted increased expression of ICAM1 in ECs of the lungs from 48h post-injection, and this increase was also abrogated in EC FAK-KO mice (Fig. 5E).

Together, these data identify EC FAK as a regulator of a cisplatin-induced secretome that promotes chemotactic SASP, vascular dysfunction with potential disruption of EC barrier and increased adhesiveness, all contributing factors to metastatic seeding in the lung.

### FAK kinase function in the nucleus regulates Cisplatin-induced DNA damage repair through ATM signaling in endothelial cells

**Figure 6:**
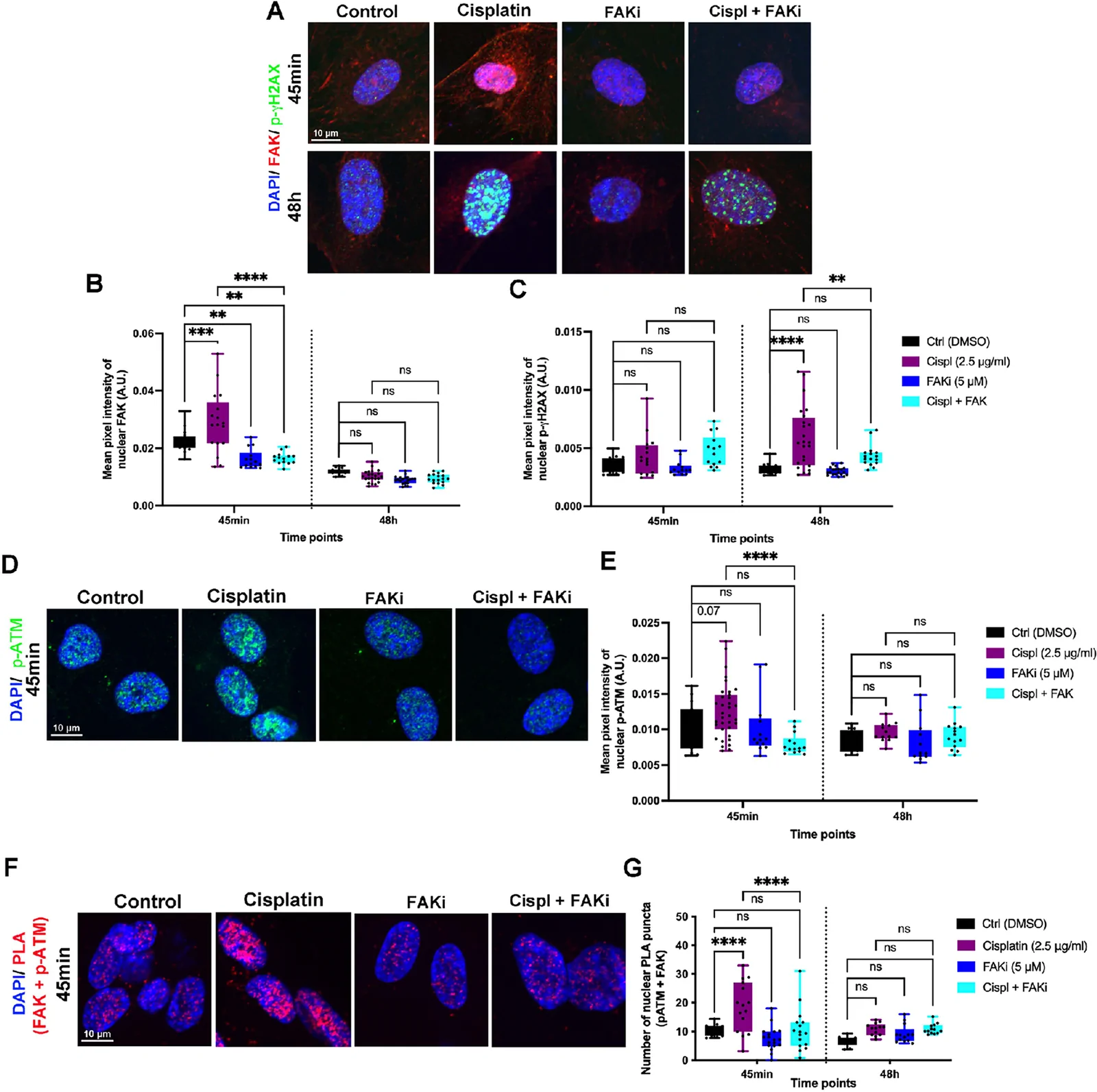
FAK transiently accumulates in the nucleus and associates with activated ATM during the early endothelial DNA damage response to cisplatin. HUVECs were treated for the corresponding timepoints with either Cisplatin, FAK inhibitor or combination. **(A)**, **(D)** and **(F)** Representative immunofluorescence images showing p-gH2AX (green), FAK (red) and DAPI (blue) **(A)**; p-ATM (green) and DAPI (blue) **(D)**; PLA (red, FAK+ p-ATM) and DAPI (blue) **(F)**. **(B), (C) and (E)** Distributions of per-field mean pixel nuclear intensities were visualized as boxplots with overlaid jittered points. A range of 12–35 fields p/condition were analysed across 2 biological replicates. **(G)** Distributions of per-field mean number of PLA nuclear puncta were visualized as boxplots with overlaid jittered points. A range of 13–18 fields p/condition were analysed across 2 biological replicates. A 2-Way ANOVA with Tukey’s multiple comparison test was performed. ns- not significant. * p<0.05; ** p<0.01; **** p<0.0001.

Beyond its cytoplasmic kinase-associated functions, FAK can also translocate to the nucleus and regulate stress-responsive transcriptional and survival pathways^75–77^. Therefore, to further investigate how EC FAK regulates cisplatin induced stress responses, we looked at the dynamics of both FAK localization and p-gH2AX in human umbilical vascular endothelial cells (HUVECs) after cisplatin treatment. Cisplatin induced a rapid and transient relocalisation of FAK to the nucleus in endothelial cells, with nuclear FAK levels peaking at 45 min and subsequently returning to baseline by 48h. Interestingly, at this 45 min time point the combined FAK kinase inhibition with Cisplatin treatment abrogated this nuclear translocation. Cisplatin treatment triggered an increase in nuclear p-gH2AX at 48h, and combined treatment abrogated this increase (Fig. 6A, C). This sequence suggests that nuclear FAK localization primes for subsequent DNA damage detected by p-gH2AX enrichment.

Although platinum-induced lesions can activate both ATR- and ATM-dependent DNA damage signalling, the endothelial transcriptional response to cisplatin was predominantly characterised by a p53-associated damage signature. This included upregulation of canonical targets such as *Cdkn1a, Mdm2, Bax, Phlda3, Trp53inp1*, and *Gdf15*, together with altered expression of DDR regulators linked to gH2AX signalling (e.g., *Ppp4r2*). In contrast, we did not detect a prominent ATR/CHK1 checkpoint transcriptional signature among the differentially expressed genes (Fig. 3E and F and **Suppl. Figure 4D and E**).

Guided by these transcriptomic findings, we investigated ATM activation and the effects of FAK kinase inhibition in HUVECs. We observed that early nuclear accumulation of FAK temporally coincided with maximal ATM activation (at 45 min-2h), as indicated by a trend in heightened nuclear pATM levels and increased pATM at 2h in whole protein lysates (Fig. 6D-E **and Suppl. Figure 6A**). This was accompanied by enhanced FAK-pATM proximity, detected by proximity ligation assay (Fig. 6F-G), suggesting a spatial association between nuclear FAK and activated ATM during the initial DNA damage response. Later (16–48 h), both nuclear and whole cell lysates pATM levels and FAK-pATM proximity returned to baseline (Fig. 6D-G **and Suppl. Figure 6A**), indicating that FAK engagement with ATM is transient and restricted to the early phase of genotoxic stress signalling. Importantly, pharmacological inhibition of FAK kinase activity significantly reduced cisplatin-induced ATM activation at early time points (45 min-2h) (Fig. 6D-G **and Suppl. Figure 6A**), supporting a role for FAK in facilitating efficient propagation of ATM-dependent DNA damage signalling.

**Figure 7:**
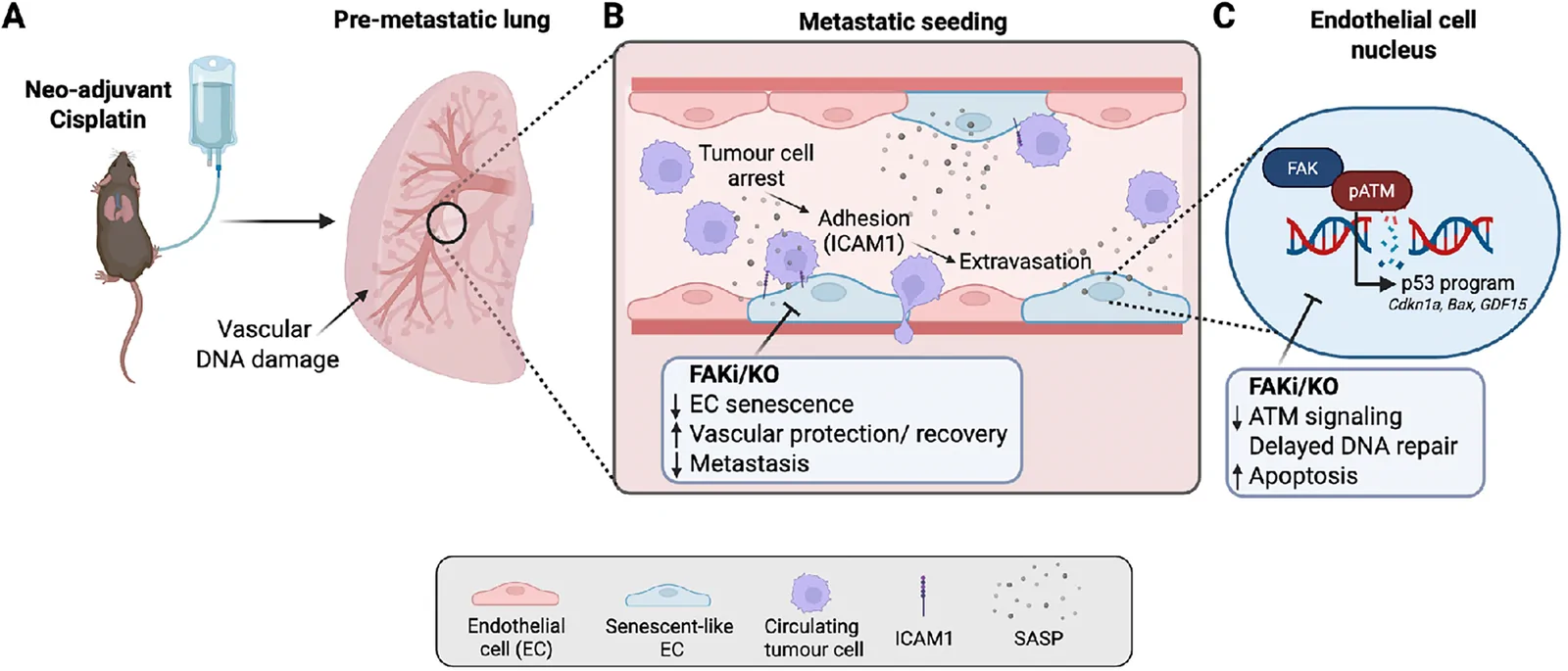
Endothelial FAK couples cisplatin-induced vascular stress responses to lung metastatic seeding. Schematic model summarizing the proposed mechanism identified in this study. Systemic cisplatin treatment induces DNA damage in vascular endothelial cells within the pre-metastatic lung vasculature, triggering early endothelial stress signaling characterized by FAK nuclear accumulation and kinase-dependent activation of ATM. This response promotes a p53-associated transcriptional signature linked to DNA damage signaling, stress adaptation, and establishment of a senescent endothelial cell state. Senescence endothelial cells acquire a pro-metastatic phenotype characterized by increased secretion of SASP-like factors, endothelial activation, matrix remodeling, and enhanced tumour cell adhesion, including increased ICAM1 expression. These changes render the lung vasculature more permissive to tumour cell arrest, seeding, and subsequent metastatic colonization. Genetic loss of endothelial FAK or pharmacological inhibition of FAK kinase activity suppresses early ATM signaling, limits endothelial senescence associated reprogramming, reduces tumour cell adhesion to the lung endothelium, and protects against cisplatin-enhanced lung metastatic seeding.

Collectively, these findings support a model in which cisplatin induces DNA damage in lung vascular endothelial cells and triggers rapid nuclear accumulation of FAK, where FAK kinase activity promotes early ATM-dependent stress signaling. This response drives a p53-associated transcriptional program and establishment of a senescent endothelial cell state characterized by altered survival, SASP-like secretion, endothelial activation, and increased tumour cell adhesion. At the tissue level, these changes convert the lung vasculature into a permissive niche for circulating tumour cells, enhancing pulmonary seeding and metastatic outgrowth (Fig. 7). Genetic loss of endothelial FAK or inhibition of its kinase activity interrupts this pathway at multiple levels, suppressing endothelial cell stress reprogramming by delaying DNA repair and forcing damaged ECs into apoptosis, thus allowing vascular recovery and protection against cisplatin-enhanced metastasis (Fig. 7).

## Discussion

In this study, we identify endothelial cell FAK as a regulator of cisplatin-induced vascular senescence that promotes metastatic seeding in the lung. We previously showed that endothelial cell FAK deletion in adult mice impaired pathological angiogenesis and suppressed tumour growth, establishing a requirement for endothelial FAK in tumour angiogenesis^33^. Subsequent work demonstrated that EC FAK loss in established tumours can also orchestrate a peri-vascular protective niche upon DNA-damaging therapy, without affecting primary tumour growth and vascular function^19^. Mechanistically, EC FAK-KO sensitized tumour cells to chemotherapy through suppression of endothelial NF-kB activation and cytokine production^19^. More recently, this paracrine protective effect was shown to be at least partially kinase dependent, with EC FAK kinase-dead (KD) mice showing similar chemosensitising abilities in Doxorubicin treated melanoma subcutaneous mouse tumour models^20^.

Previous studies have shown that endothelial expression of kinase-dead FAK (EC FAK-KD) reduces spontaneous and experimental metastasis by preserving vascular barrier integrity downstream of VEGF and VE-cadherin signalling, thereby limiting tumour cell extravasation^78^. However, in the experimental setting tested here, neither EC FAK deletion nor EC FAK-KD alone reduced lung metastasis under saline-treated conditions when compared with their respective controls. A protective effect was only revealed following neo-adjuvant cisplatin treatment. Several differences between our study and that of Jean *et al.*^78^ may explain these divergent phenotypes in untreated mice. First, Jean et al. used the 5′ endothelial enhancer of the stem cell leukaemia (SCL) locus to drive FAK-KD expression, whereas our model uses a Pdgfb-driven endothelial Cre system. Second, their study analysed heterozygous FAK-KD mice, while we used homozygous FAK-KD animals. Third, their conclusions were based on B16F10 melanoma, 4T1 breast cancer and ID8 ovarian cancer models, whereas our study focused specifically on lung cancer cell lines. Thus, while the previous study established a role for endothelial FAK kinase activity in regulating vascular permeability and tumour cell extravasation across several tumour types, our data indicate that, in lung cancer metastasis, endothelial FAK becomes functionally important primarily in the context of chemotherapy-induced vascular stress.

More recently, we reported that endothelial FAK loss reduced liver and lung metastasis following gemcitabine treatment in pancreatic ductal adenocarcinoma, and that low endothelial FAK expression was associated with improved outcome in treated patients^31^. In that study, however, endothelial cell FAK deletion did not alter circulating tumour cell numbers, tumour cell homing or early seeding after intrasplenic injection of PDAC cells following neo-adjuvant gemcitabine treatment, suggesting that endothelial cell FAK mainly regulated later stages of metastatic colonisation in the liver^31^. In contrast, the present study shows that EC FAK deletion abrogates the cisplatin-driven increase in multiple stages of lung metastasis, through a DNA damage- and stress-induced response in pre-metastatic lung vascular endothelial cells. Together, these findings suggest that endothelial FAK has context-dependent and separable functions in vascular permeability, angiogenesis initiation, vascular remodelling and therapy-induced angiocrine signalling. The dominant metastatic phenotype appears to depend on tumour type, target organ, timing and strength of endothelial perturbation and the specific treatment setting.

We have also observed a protective effect of both EC FAK-KO and EC FAK-KD on the cisplatin treated primary tumour vasculature, rescuing vessel numbers and perfusion. Therefore, we cannot fully exclude that, in the presence of a primary tumour, cisplatin induced vascular changes in the primary tumour might also contribute to the increase of lung metastasis observed. However, our *in vivo* intravenous metastasis experiments further show that the endothelial changes caused by cisplatin treatment, are sufficient to condition the lung independently of the primary tumour. These data separate host conditioning from continued primary tumour-derived signaling and indicate that chemotherapy can generate a metastasis-permissive pulmonary vascular state on its own. Consistent with this, we also observed increased percentage of ICAM1 positive ECs with Cisplatin treatment, which was abrogated in EC FAK-KO mice. Accordingly, *in vitro*, we not only observed increased adhesion of tumour cells to pre-treated human ECs with cisplatin treatment, but we also identified a set of cisplatin-induced proteins linked to proteolysis, endothelial activation, matrix remodeling, and adhesive interactions^59^, all of which were consistently suppressed by pharmacological FAK inhibition. Together, the *in vivo* ICAM1 increase and the plethora of *in vitro* uniquely secreted factors upon Cisplatin treatment, provide plausible downstream effectors linking FAK dependent DNA-damage endothelial stress reprogramming to increased tumour cell adhesion and transmigration through the lung vasculature^73,74^, and suggesting these are at least partially kinase dependent.

A key observation of this study is that cisplatin induces a senescent endothelial cell phenotype both *in vitro* and in the pre-metastatic lung *in vivo*. This fits with the broader concept that therapy-induced senescence is not restricted to tumour cells but can be triggered in stromal compartments, supporting resistance, relapse and metastasis^25,26^. Although vascular endothelial cell senescence has been linked to lung metastasis^29^, here we show that cisplatin induces endothelial cell senescence in the pre-metastatic lungs, promoting metastasis development. Our data further indicate that endothelial cell FAK and its kinase activity are required for efficient establishment of this chemotherapy-induced endothelial stress state. In EC FAK-KO mice, cisplatin-induced endothelial p16 reporter activity in the pre-metastatic lung was reduced, and *in vitro* FAK kinase inhibition attenuated senescence-associated features after cisplatin exposure. Furthermore, in cisplatin-treated control mice, pre-metastatic ECs upregulated a p53- and stress-associated signature consistent with a coordinated genotoxic stress response with features of growth arrest and validated by increased p-γH2Ax *in vivo*. This stress-response was attenuated in EC FAK-KO mice, placing FAK upstream of endothelial cell adaptation to cisplatin *in vivo*. At the same time, combined cisplatin and FAK pharmacological inhibition *in vitro*, reduced endothelial cell viability and increased apoptosis relative to either treatment alone, raising the possibility that FAK activity supports survival of damaged vascular endothelial cells and thereby permits persistence of a dysfunctional senescent endothelial cell population.

Our findings identify a previously unrecognised role for endothelial cell FAK in coordinating the early nuclear DNA damage response to cisplatin. We show that FAK undergoes rapid and transient nuclear accumulation following cisplatin exposure, coinciding with ATM activation and increased spatial proximity between FAK and phosphorylated ATM. This temporally restricted interaction suggests that nuclear FAK may contribute to the early assembly or stabilisation of ATM signalling complexes, thereby facilitating efficient propagation of the DNA damage response. Consistent with this, inhibition of FAK kinase activity attenuated ATM activation and reduced the delayed accumulation of gH2AX, indicating that FAK is required for full amplification of ATM-dependent signalling. Moreover, endothelial cell FAK deficiency impaired the induction of canonical p53-dependent checkpoint and DNA repair genes, further supporting a role for FAK in coordinating early genotoxic stress responses.

These findings are consistent with previous reports showing that FAK can translocate to the nucleus and regulate stress-responsive transcriptional and survival pathways^75^. For example, nuclear FAK has been shown to control inflammatory VCAM1 expression through regulation of GATA4 stability, highlighting a non-canonical nuclear function in vascular endothelial cells during development^79^. In other contexts, nuclear FAK can bind p53 via its FERM domain and promote MDM2-dependent p53 ubiquitination and degradation, thereby supporting cell survival under stress^80,81^. Although we do not directly demonstrate formation of a FAK–MDM2–p53 complex in endothelial cells, our data are in line with this model: endothelial FAK deficiency reduced expression of the p53 target *Mdm2*, while increasing expression of the p53 regulator *Mdm4*^56^. In addition, FAK has been implicated in promoting DNA repair following ionising radiation in oncogenic KRAS-driven NSCLC, where its inhibition delays repair and enhances DNA damage accumulation^23^. More recently, nuclear and kinase-active FAK has been associated with resistance to cisplatin in high-grade serous ovarian cancer, where it supports tumour cell survival following chemotherapy^77^.

Together, these observations suggest that nuclear FAK plays a broader role in determining cell fate following genotoxic stress. In the endothelial cell context, our findings support a model in which FAK influences whether cisplatin-damaged cells undergo transient repair, persist in a senescent state, or are eliminated through apoptosis.

This study has several implications. First, it identifies the lung endothelium as a target of platinum-induced injury with consequences for metastatic progression. Second, it places endothelial cell FAK at the intersection of acute DNA damage signaling and longer-term vascular lung niche remodeling. Third, it suggests that the pro-metastatic effects of chemotherapy can arise not only from tumour cell selection, but also from maladaptive vascular endothelial cell stress responses. This raises the broader question of whether more tumour-selective delivery of cytotoxic agents could reduce therapy-induced damage to distant vascular niches. In this context, tumour-targeted or organ-sparing formulations of platinum-based chemotherapy may represent one strategy to preserve anti-tumour efficacy while limiting systemic vascular injury and metastatic conditioning.

Our findings also support further investigation of endothelial cell FAK targeting as a strategy to limit chemotherapy-induced metastatic conditioning, particularly because FAK inhibitors are already being clinically tested in combination with anti-cancer therapies^18^. Nevertheless, systemic FAK inhibition may also have unwanted effects in other vascular beds or stromal compartments, given the broad role of FAK in endothelial cell homeostasis, tissue repair, immune regulation and mechanotransduction^18^. Future therapeutic strategies may therefore require more selective targeting of FAK-dependent stress responses in the tumour-bearing lung or pre-metastatic niche, rather than complete systemic pathway inhibition.

More broadly, this data also suggests that senescence-directed interventions in cancer therapy may need to consider vascular endothelial cells in addition to tumour cells and fibroblastic stromal populations. Defining robust biomarkers of therapy-induced endothelial cell senescence, and determining when and where these emerge after chemotherapy, will be essential for identifying patients most likely to benefit from senomorphic, senolytic or vascular-protective combination strategies. In summary, our findings provide a mechanistic framework linking chemotherapy-induced vascular endothelial cell injury to pre-metastatic niche formation and identify endothelial cell FAK as a candidate target for limiting the unintended pro-metastatic effects of cytotoxic therapy.

## Supplementary Material

Supplementary Files

This is a list of supplementary files associated with this preprint. Click to download.

• Supplfigurestosubmit.pdf

## Data Availability

The RNA-seq data underlying figure 3E-F and Suppl. figure 4B-E are openly available in GEO, under the accession number GSE328693. The mass spectrometry proteomics data underlying figure 5A have been deposited to the ProteomeXchange Consortium via the PRIDE^82^ partner repository with the dataset identifier PXD079223 and 10.6019/PXD079223.
